# Peperomin E and its orally bioavailable analog induce oxidative stress-mediated apoptosis of acute myeloid leukemia progenitor cells by targeting thioredoxin reductase

**DOI:** 10.1016/j.redox.2019.101153

**Published:** 2019-03-08

**Authors:** Xinzhi Wang, Ming Gao, Jiyun Zhang, Ying Ma, Wenshu Qu, Jingyu Liang, Hao Wu, Hongmei Wen

**Affiliations:** a School of Pharmacy, Nanjing University of Chinese Medicine, Xianlin Avenue No. 138, Nanjing 210023, People's Republic of China; b Nanjing University of Science and Technology Hospital, Nanjing University of Science and Technology, Xiaolinwei Lane No. 200, Nanjing 210094, People's Republic of China; c People's Liberation Army Cancer Center, Nanjing Bayi Hospital, Yanggongjing Street No. 34, Nanjing 210002, People's Republic of China; d Department of Natural Medicinal Chemistry, China Pharmaceutical University, Tongjia Lane No.24, Nanjing 210009, People's Republic of China

**Keywords:** Peperomin E, Orally bioavailable analog, Acute myeloid leukemia progenitor cells, Oxidative-mediated apoptosis, Thioredoxin reductase 1

## Abstract

The early immature CD34^+^ acute myeloid leukemia (AML) cell subpopulation-acute myeloid leukemia progenitor cells (APCs), is often resistant to conventional chemotherapy, making them largely responsible for the relapse of AML. However, to date, the eradication of APCs remains a major challenge. We previously reported a naturally occurring secolignan- Peperomin E (PepE) and its analog 6-methyl (hydroxyethyl) amino-2, 6-dihydropeperomin E (DMAPE) that selectively target and induce oxidative stress-mediated apoptosis in KG-1a CD34^+^ cells (an APCs-like cell line) in vitro. We therefore further evaluated the efficacy and the mechanism of action of these compounds in this study. We found that PepE and DMAPE have similar potential to eliminate primary APCs, with no substantial toxicities to the normal cells *in vitro* and *in vivo*. Mechanistically, these agents selectively inhibit TrxR1, an antioxidant enzyme aberrantly expressed in APCs, by covalently binding to its selenocysteine residue at the *C*-terminal redox center. TrxR1 inhibition mediated by PepE (DMAPE) leads to the formation of cellular selenium compromised thioredoxin reductase-derived apoptotic protein (SecTRAP), oxidation of Trx, induction of oxidative stress and finally activation of apoptosis of APCs. Our results demonstrate a potential anti-APCs molecular target – TrxR1 and provide valuable insights into the mechanism underlying PepE (DMAPE)-induced cytotoxicity of APCs, and support the further preclinical investigations on PepE (DMAPE)-related therapies for the treatment of relapsed AML.

## Graphical abstract

**Figure:**
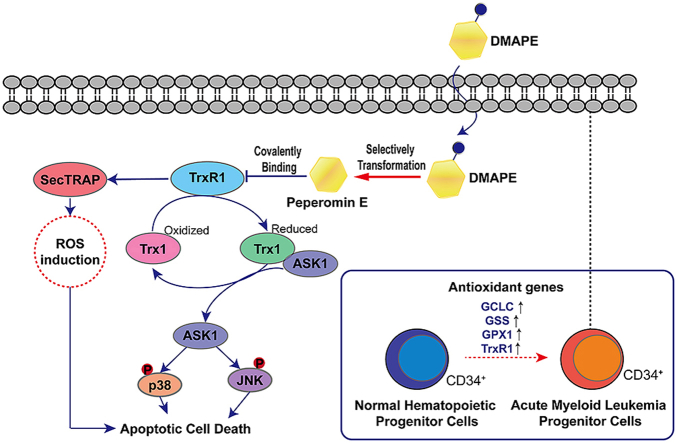

## 1 Introduction

Acute myeloid leukemia (AML) is a malignant disease characterized by an aberrant accumulation of immature myeloid hematopoietic cells [1]. The combination of an anthracycline and cytarabine (Ara-C) is considered as the gold standard induction therapy for patients with AML, resulting in complete remission rates of 50–75% [2,3]. Although remission can be achieved in most patients, disease relapse is common and long-term survival is poor in most cases [4]. CD34 is a stage-specific antigen that is expressed on human hematopoietic stem and progenitor cells [5,6]. Recent studies have shown that the early immature CD34^+^ AML cell population is frequently impervious to the conventional chemotherapy, making them largely responsible for clinical relapse of the disease. Moreover, the current chemotherapy agents may not effectively discriminate between normal and malignant cells [[7], [8], [9]]. For this reason, it is crucial to identify therapies that can specifically target the CD34^+^ AML population while sparing normal cells.

However, identifying CD34^+^ AML cell-targeting therapeutic agents is challenging. CD34^+^ AML cells shares similar stem-like characteristics with CD34^+^ normal bone marrow cells [[8], [9], [10]]. The unique features that render CD34^+^ AML cells as a potential risk factor of hematological malignancies are still under debate. Recent studies suggested that the aberrant regulation of signaling pathways such as ROS, NF-κB, Wnt/β-catenin, or Hedgehog pathway is critical to the pathogenesis of CD34^+^ AML cells [[11], [12], [13], [14], [15], [16], [17]]. The recovery of these signaling pathways could be used for the elimination of CD34^+^ AML cells. Therefore, agents that inhibit these signaling pathways have been tested recently and some have been proven to target CD34^+^ AML cells [[18], [19], [20], [21], [22]]. These reported CD34^+^ AML cell-targeting agents, however, are still at early stages of development; most of them have relatively poor pharmacologic properties and are not suitable for clinical use [11,12]. Thus, searching for new targets and novel therapeutic agents to eradicate the CD34^+^ AML cells remains a major task for the researchers.

**Fig. 1:**
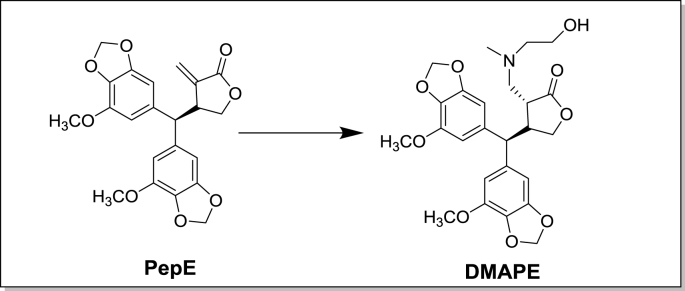
The chemical structures of PepE and DMAPE.

In our previous work, a naturally occurring secoligan – Peperomin E (PepE, Fig. 1) isolated from a commonly used anti-cancer folk medicine *Peperomia dindygulensis* in China, was found to effectively target and initiate oxidative stress mediated apoptosis in CD34^+^ AML cells with no apparent toxicity to normal cells in vitro [23]. However, despite its high efficacy in vitro, the solubility and oral bioavailability of PepE are relatively poor, making its pharmaceutical use extremely challenging. Therefore, we synthesized and screened a series of amino-derivatives of PepE to identify a compound with improved solubility and bioavailability. We generated an *N*-methyl ethanolamine analog, 6-methyl (hydroxyethyl) amino-2, 6-dihydropeperomin E (DMAPE, Fig. 1), which when formulated with phosphate acid, demonstrated more than 1000-fold increased solubility in water compared to PepE. Most importantly, preliminary in vitro studies indicated that DMAPE can also selectively eradicate CD34^+^ AML cells similar to PepE without apparent toxicity to normal bone marrow cells, making it a novel drug lead for CD34^+^ AML cell-targeted therapy [23].

Although the in vitro efficacy of PepE (DMAPE) has been demonstrated, their *in vivo* therapeutic efficacy, primary molecular target, and mode of action remain unclear. The aim of the present work was to evaluate the potential use of PepE (DMAPE) as a CD34^+^ AML cell-targeted therapy. Therefore, the effects of PepE (DAMPE) on primary CD34^+^ hematopoietic cells isolated from AML patients, and in a humanized murine model of leukemia, were investigated. Furthermore, we sought to elucidate the molecular target and mechanisms by which PepE (DMAPE) functions to induce oxidative stress mediated apoptosis in CD34^+^ AML cells.

## 2 Materials and methods

### 2.1 Materials

Peperomin E (PepE) and Peperomin A (PepA) were isolated in our laboratory through a series of chromatographic procedures from *P. dindygulensis*. (2*S*,3*S*)-6-Methyl (hydroxyethyl) amino-2,6-dihydropeperomin E (DMAPE) phosphate salt was synthesized in our laboratory following a previously published procedure [23]. The chemical structures were unequivocally elucidated using multiple spectroscopic methods (Figs. S1–3). The purity of all chemicals was verified by high-performance liquid chromatography peak area normalization and peak purity analysis. Results showed that the purity was >98% and the peak purity angle/peak purity threshold was <1 for these compounds.

Parthenolide was purchased from Cayman Chemical (Ann Arbor, MI, USA). Cytarabine (Ara-C) was obtained from Abmole Bioscience Inc. (Houston, TX, USA). (−)-Arctigenin, D-luciferin phosphate, and dihydroethidium (DHE) were purchased from Santa Cruz Biotech (Santa Cruz, USA). 2′,7′-dichlorofluorescein diacetate (DCFH-DA), N-acetyl-l-cysteine (NAC), 5,5′-dithiobis (2-nitrobenzoic acid) (DTNB), 1-chloro-2,4-dinitrobenzene (DNCB), 2,3-dimercapto-1-propanesulfonic (DMPS), diamide, tris(2-carboxyethyl) phosphine hydrochloride (TCEP), ethylene glycol-bis(2-aminoethyl ether)-N,N,N′,N'-tetraacetic acid (EGTA), dimethyl sulfoxide (DMSO), phenylarsine oxide (PAO), *β*-Nicotinamide adenine dinucleotide 2′-phosphate reduced tetrasodium salt hydrate (NADPH), Iodoacetamide (IAA), protein G-Sepharose, and Sepharose 4B were obtained from Sigma-Aldrich (St. Louis, USA). N-(Biotinoyl)-N'-(Iodoacetyl) Ethylenediamine (BIAM), horseradish peroxidase (HRP)-conjugated streptavidin were obtained from Thermo-Fisher Scientific (Rockford, USA) The TrxR-responding fluorescence probe TR-green synthesized by Huang's method [25] was kindly provided by Prof. Wei Li, Nanjing University of Chinese Medicine.

Rabbit monoclonal anti-human Superoxide dismutase 1 & 2 (SOD1&2), Glutamate cysteine ligase catalytic subunit (GCLC), Glutathione synthetase (GSS), Catalase (CAT), Glutathione peroxidase 1 (GPX1), Glutathione reductase (GSR), Peroxiredoxin 1 (PRX1), and Thioredoxin reductase 1(TXNRD1, also known as TrxR1); recombinant human TXNRD1 (full length, contains the complete *C*-terminal peptide sequence –SGASILQAGCUG-, product No. ab204206), TXNRD1 (protein fragment, without selenocysteine residue, *C*-terminal peptide sequence is –SGASILQAGC-, product No. ab168011), GPX1, GSS, and GCLC proteins were all purchased from Abcam (San Francisco, CA, USA). Rabbit polyclonal antibodies against GAPDH, *β*-actin, Thioredoxin 1 (Trx1), Apoptosis signal-regulating kinase 1 (ASK1), c-Jun N-terminal kinase (JNK), and its phosphorylated-form (Thr183/Tyr185), p38 mitogen-activated protein kinases (MAPK) and its phosphorylated-form (Thr180/Tyr182), full-length and cleaved poly ADP-ribose polymerase (PARP), and full-length and cleaved caspase 3 were purchased from Cell Signaling Technology Inc. (Beverly, MA, USA). Horseradish peroxidase (HRP)-conjugated secondary antibody (goat anti-rabbit), human TrxR1 shRNA plasmids, control shRNA plasmids, TrxR1 CRISPR activation plasmids, control CRISPR activation plasmids were obtained from Santa Cruz Biotech. Gibco fetal bovine serum (FBS), Iscove's Modified Dulbecco's Medium (IMDM) cultural media, phosphate buffer saline (PBS), and penicillin-streptomycin solution were purchased from Thermo-Fisher Scientific. The Polyvinylidene fluoride (PVDF) transfer membrane (0.2 μm) was obtained from Merck Millipore Ltd. (Carringtwohill, Ireland). Phenylmethylsulfonyl fluoride (PMSF), bovine serum albumin (BSA), Radio-immunoprecipitation assay (RIPA) buffer, and Cell counting kit-8 (CCK-8) were purchased from Solarbio Ltd. (Beijing, China). The apoptosis detection kit containing fluorescein-5-isothiocyanate-conjugated Annexin V (Annexin V-FITC) and Propidium iodide (PI) was from Beyotime (Nantong, China). Magnetic-activated cell sorting (MACS) CD34 MicroBead kit, anti-FITC microbeads, and monoclonal anti-human CD34-FITC antibodies were purchased from Miltenyi Biotec (Teterow, Germany).

### 2.2 Human specimens and culture

KG-1a cell line had a large portion of cells bearing a CD34^+^ immunophenotype which was considered to be characteristic of APCs. Therefore, this cell line was considered to be an APCs-like cell line for a preliminary screening of potential anti-APCs agents [21]. This cell line were previously purchased from American Type Culture Collection (ATCC, Manassas, USA). Human bone marrow stem cells (hBMSC, product No. BNCC341707) was previously obtained from BeNa Culture Collection Ltd. (Beijing, China).

Primary human AML samples and normal bone marrow cells (NBM) were obtained from volunteer donors from Jiangsu Province Hospital on Integration of Chinese and Western Medicine (Nanjing, China). The clinical information of AML specimens are detailed in Table S1. Mononuclear cells were isolated from the samples using Ficoll-Plaque (GE healthcare, USA) density gradient separation and cryopreserved at −80 °C in a freezing medium of 90% FBS and 10% DMSO until use. All cells were cultured in IMDM-based complete medium, consisting of 90% IMDM medium and 10% FBS, supplemented with 100 U/mL penicillin and 100 μg/ml streptomycin in 25 cm^2^ flasks (Corning, New York, USA) at 37 °C in a 5% CO_2_ incubator (Thermo Scientific, Massachusetts, USA).

### 2.3 Progenitor cell isolation and treatment

The mononuclear cells were further enriched for CD34 positive cells using the MACS CD34 MicroBead kit following the manufacturer's protocol. Enriched CD34 cell-purity was measured by flow cytometry-based quantification of cells stained using an anti-CD34-FITC antigen. Isolated CD34^+^ cells were pre-incubated in the IMDM-based serum-free media for 1 h before the addition of drugs. PepE was dissolved in DMSO to make a 100 mM stock solution and stored in aliquots at – 20 °C. The 100 mM stock solution was diluted with DMSO to 10 mM and then diluted with PBS to 1 mM before treating the cells at the indicated concentrations. DMAPE and Ara-C were diluted in PBS to 1 mM concentration before use.

### 2.4 Cell viability assay

Unless otherwise mentioned, 2 × 10^4^ cells were seeded at 96-well plates (Corning, New York, USA) in the IMDM-based serum-free media for 1 h. Then, the cells were incubated with different concentrations of PepE and DMAPE at 37 °C in a 5% CO_2_ incubator for 48 h. At the end of the treatment, 10 μL CCK-8 solution was added to each well and incubated for an additional 2 h at 37 °C, and the absorbance at 450 nm was measured with a microplate reader (Thermo Scientific, Multiskan GO, Finland). Inhibition ratio (%) was calculated as [(absorbance of control − absorbance of the test sample)/absorbance of control] x 100%.

### 2.5 Pharmacokinetic study of DMAPE *in vivo*

The UFLC-MS/MS system consisted of a Prominence™ UFLC system (Shimadzu, Kyoto, Japan) coupled with a QTRAP™ 5500 MS/MS system (Applied Biosystems/MDS SCIEX, Foster City, CA, USA) and controlled by the Analyst 1.5.2 software. MS‐grade methanol and acetonitrile were purchased from Merck (Darmstadt, Germany). Ultra‐pure water was generated using a MilliQ™ direct water purification system (Merck–Millipore, Billerica, MA, USA). The mass spectra and chromatographic conditions, method validations, standard and sample preparation for simultaneous determination of DMAPE and its parent drug PepE in rat plasma are provided in the *Additional method in* Supplementary Materials.

Male Sprague–Dawley rats weighing 200 ± 20 g were supplied by the Qinglongshan Experimental Animal Breeding Company (Nanjing, China). The experiment protocol was approved by the Animal Ethics Committee of Nanjing University of Chinese Medicine (Nanjing, China). The rats were housed under controlled environmental conditions (temperature 25 ± 2 °C, relative humidity 50 ± 10%) with free access to standard laboratory food and water. Before drug administration, the rats were randomly divided into two groups (eight rats per group) and fasted for 12 h with access to water. DMAPE phosphate salt aqueous solution was i. g. (50 mg/kg) or i. v. (50 mg/kg) administrated. Blood samples were collected from the tail vein into heparinized Eppendorf tubes using lithium heparin‐containing microvettes. The plasma was obtained by centrifugation of the blood at 5000 rpm for 10 min and then frozen at −20 °C until analysis. Pharmacokinetic parameters were calculated using the DAS 2.1.1 software (Pharmacological Society of China, China) using non-compartmental analysis.

### 2.6 Release study of DMAPE in vitro

1 × 10^5^ cells (including sorted CD34^+^ KG-1a cells and NBM cells) were seeded at 48-well plates (Corning, New York, USA) in the IMDM-based serum-free media for 1 h. The cells were then incubated with 10 μM DMAPE at 37 °C in the 5% CO_2_ incubator for 0, 12 or 24 h. The culture supernatant was collected by centrifugation at 3000 rpm for 10 min and evaporated to dryness at 30 °C using a CentriVap vacuum centrifugal concentrator. The resulting residue was reconstituted in 100 μL methanol, vortexed and centrifuged at 12000 rpm for 10 min. A 2 μL aliquot of the supernatant was injected for UFLC-MS/MS analysis (UFLC-MS/MS analysis method same as the pharmacokinetic study of DMAPE, see *Additional method in* Supplementary Materials).

### 2.7 NOD/SCID mouse assays

Six-week-old female NOD-SCID mice (obtained from the model animal research center of Nanjing University, Nanjing, China) were acclimated for 1 week and housed in groups of five. All animal studies were conducted under the guidelines approved by the Animal Care and Use Committee of the Nanjing University of Chinese Medicine (Nanjing, China). The mice were sublethally irradiated with 2.7 Gy before transplantation.

KG-1a cells tagged with a luciferase reporter gene (KG-1a-Luc) were obtained from Cobioer biotechnology Inc. (Nanjing, Jiangsu, China) and cultured in 90% IMDM supplemented with 10% FBS, 0.05 mM 2-Mercaptoethanol, and 1 μg/mL Puromycin, and grown at 37 °C in a 5% CO_2_ humidified incubator. CD34 positive cells were enriched (following the 2.3 method) and injected via the tail vein in a final volume of 0.2 mL PBS with 0.5% FBS. For analyzing the luciferase signal, 5 × 10^6^ cells KG-1a-Luc cells were used for each recipient animal. Seven days after transplantation, mice with KG-1a-Luc implants were subjected to bioluminescent analysis based on the luciferase signal. Briefly, mice received D-luciferin phosphate solution (100 mg/kg) intravenously 10 min prior to imaging. They were then anesthetized with isoflurane inhalation and imaged using a Xenogen IVIS scanner (PerkinElmer, Waltham, MA, USA). The next day, mice showing successful implantations were randomly divided into the control, DMAPE low dose, DMAPE high dose, and Ara-C treatment groups (n = 5 in each group), which received daily i. g. saline buffer, DMAPE phosphate salt 25 mg/kg, DMAPE phosphate salt 50 mg/kg and Ara-C (50 mg/kg), respectively. The growth of KG-1a-Luc cells was monitored every week by their luciferase signal. Five weeks after the implantation, all mice were sacrificed and their livers, kidneys, and bone marrow were excised for *ex vivo* bioluminescent imaging. The bioluminescent signal intensity was all quantified using the Living Image software (version 4.2, Carliper Life Science, Inc., Hopkinton, MA, USA) and is presented as photons/second/cm^2^/sr (sr denotes steradian).

### 2.8 Apoptosis assay

KG-1a CD34^+^ and other sorted primary APCs (1 × 10^6^) were incubated with 6 μM PepE or DMAPE in the presence or absence of 5 mM NAC for 24 h in 6-well plates (Corning), respectively. The cells were harvested and washed twice with PBS. The apoptotic cells, necrotic cells, and live cells were identified by PI and Annexin V-FITC staining assay following the manufacturer's instructions. Data were obtained and analyzed using a BD Accuri™ C6 flow cytometer (BD Biosciences, San Jose, CA, USA) with CellQuest software (BD Biosciences).

### 2.9 Intracellular ROS measurement

KG-1a CD34^+^ cells and other sorted primary APCs (5 × 10^5^) were plated in FBS-free IMDM medium in 12-well plates (Corning) and were treated with 5 μM of Ara-C and 6 μM PepE or DMAPE in the presence or absence of 5 mM NAC for 2 h. The ROS indicator DCFH-DA (10 μM) or DHE (10 μM) in fresh FBS-free medium was added to each well, and further incubated in the dark for 30 min at 37 °C. The cells were visualized and photographed under an Olympus inverted fluorescence microscope IX-73 (Tokyo, Japan) with Metamorph software (Molecular Devices, Downingtown, PA, USA).

### 2.10 Western blot analysis

For western blot analysis, total cellular proteins were extracted by RIPA + PMSF (100:1) buffer and were quantified using the Bradford procedure. Equal amounts of protein in each sample lysate were separated by SDS-PAGE under reducing conditions and then transferred to PVDF membranes. The blots were then blocked with 5% BSA in TBST at room temperature for 1 h. The membranes were then incubated with specific primary antibodies in 5% BSA at 4 °C for 12 h. Following five washes with TBST, the membranes were incubated with HRP-conjugated secondary antibodies for 1 h at room temperature, washed with TBST five times and transferred to freshly made ECL solution (Yeasen Biotech, Shanghai, China). The immune-reactive bands were visualized under Tanon 5200 chemiluminescence imaging analysis system (Shanghai, China) and analyzed using Gel-pro 32 software (Media Cybernetics, Rockville, MD, USA).

### 2.11 Quantitative real-time reverse transcription PCR (qRT-PCR)

Total mRNA from the cells was isolated with the RNeasy Midi-kit (Qiagen, Valencia, CA, USA) following the manufacturer's instructions. The purity and quantity of mRNA were determined by NanoDrop (Thermo). mRNA samples were reserve transcribed into cDNA using the TransScript One-Step RT-PCR SuperMix kit (Transgen Biotech, Beijing, China). RT-PCR was performed with Applied Biosystems 7500 RT-PCR system (Thermo) using PowerUp SYBR Green Master Mix reagent (Thermo). Expression of each gene was first internally normalized to the mean expression of human HPRT1 gene. The average expression of each gene in CD34^+^ NBM cells (n = 3) was set to 1, and the relative expression of each gene in each sample was calculated accordingly. To determine the knockdown/activate efficiency, expression of TrxR1 was first internally normalized to GAPDH and then used for comparison. Primer sequences for qRT-PCR are listed in Table S2.

### 2.12 Bio-layer interferometry (BLI) binding assay

The binding kinetics of PepE or PepA to purified recombinant proteins were determined using BLI on an Octet RED 96 system (ForteBio, Shanghai, China) following the manufacturer's protocol. All interaction analyses were performed at 25 °C in PBS with 0.2% DMSO. Purified recombinant proteins (0.9 μM, the TrxR1 full length and TrxR1 protein fragment were pre-reduced by 400 μM of NADPH before test) were pre-labeled with biotin using streptavidin (SA) conjugation kit (Abcam) following the manufacturer's instruction. Loading of the Dip and Read Super Streptavidin (SSA) Biosensors (ForteBio) were conducted by exposing biotinylated proteins to biosensor tips for 2 h. The 96-well microplates used in the Octet were filled with 200 μL sample or buffer per well and agitated at 1000 rpm. The loaded biosensors were washed in buffer for 600 s and transferred to wells containing PepE at concentrations of 0.625, 1.25, 2.5, 5, and 10 μM or PepA at concentrations of 1.25, 2.5, 5, 10 and 20 μM, respectively, in the buffer. The association and dissociation were observed for 90 s for each sample diluent. Reference measurements were conducted using the buffer instead of PepE (or PepA). A parallel set of SA unloaded biosensors was prepared to act as a control. The sample-sensorgrams were corrected by subtracting the double reference curve. Kinetic parameters (K_on_ and K_dis_) and affinity (K_D_) were determined from a global fit to a 1:1 binding model of the data between proteins and PepE using Octet software (ForteBio).

### 2.13 In vitro TrxR1 activity assay

The in vitro TrxR1 activity assay was performed using the DTNB reduction assay [24]. Briefly, 0.1 μM recombinant human TrxR1 or TrxR1 without Sec498 residue (TrxR1 nSec) protein was reduced with 100 μM NADPH in assay buffer (50 mM Tris-HCl, 1 mM EDTA, pH = 7.5) for 30 min at room temperature. The reduced proteins was then treated with the indicated concentrations of PepE (final concentrations of 0.25, 0.5, 1, 2, 4, 6 μM), respectively, and incubated for 1 h at 37 °C. A mixture of assay buffer solution (50 mM Tris-HCl, 1 mM EDTA, pH = 7.5) containing DTNB and NADPH was added then added, making the final concentrations of DTNB and NADPH 2 mM and 200 μM, respectively. The absorbance was monitored at 412 nm using the SpectraMax i3x microplate reader (Molecular Devices). The linear increase of absorbance during the initial 3 min was recorded for calculating the relative enzyme activity. The same amount of DMSO was added to the control experiments and the activity is expressed as the percentage of the control.

### 2.14 Molecular modeling

Covalent docking of PepE and TrxR1 was performed in the Schrödinger Software Suite (V2015-2, Schrödinger LLC, NY, USA). The structure of human TrxR1 protein (PDB ID: 2J3N, Chain A, B, C, D, E, and F) was obtained from the Protein Data Bank and further modified in the Protein Preparation Wizard module. The Sec498 residue in chain C was selected as the reactive residue involved in the Michael addition and also set as the centroid of the docking box (Box size: dock ligands with length ≤ 15 Å). The other dock parameters were set to default in the docking simulation.

### 2.15 The BIAM labeling assay

To further assess whether the Sec residue in TrxR1 was the modified site of PepE. The BIAM labeling assay was performed according to previous report [26,27]. Briefly, 100 μM BIAM stock solution in 100 mM Tris-HCl, 1 mM EDTA buffer (pH = 6.5) was freshly prepared before each test. The TrxR1 protein (1 μM) was incubated with different concentrations (1, 5, 10 μM) of PepE or 5 μM of DNCB (positive control) and DMSO (negative control) in 50 mM Tris-HCl, 1 mM EDTA buffer (pH = 7.5) and 400 μM NADPH at 37 °C. After incubation, 4 μL of the reaction mixture was withdrawn and added mixed with 20 μM of BIAM solution in 100 mM Tris-HCl, 1 mM EDTA assay buffer (pH = 6.5) for 30 min at 37 °C to allow selective alkylation of free Sec residues. The reaction was stopped by adding the IAA solution (final concentration 50 mM). The samples were then denatured in SDS buffer, boiled and subjected to SDS-PAGE and western-blotting on a 7.5% gel, and the separated proteins were transferred to nitrocellulose membrane. The enzymes labeled with BIAM were detected with HRP-conjugated streptavidin and enhanced chemiluminescence detection. The bands were analyzed using Gel-pro 32 software.

### 2.16 The NADPH oxidative activity assay

The NADPH oxidative activity by TrxR1 in the presence of PepE was performed according to the previous reports [26,28]. Briefly, the recombinant human TrxR1 protein (0.2 μM) was incubated with either 5 μM of PepE or 5 μM of DNCB at room temperature in assay buffer (50 mM Tris-HCl, 1 mM EDTA, pH = 7.5), same amount of DMSO was used as negative control. NADPH was then added (final concentration was set at 200 μM) and the absorbance was monitored at 340 nm for 30 min using the SpectraMax i3x microplate reader (Molecular Devices), the molar extinction coefficient was set as 6200 M^−1^cm^−1^. The linear decrease in NADPH absorbance was used for calculating NADPH consumption rate and expressed as NADPH μM^−1^min^−1^enzyme μM^−1^.

### 2.17 Imaging TrxR1 activity in cells

The specific fluorescence probe of TrxR – TR-green was employed to image TrxR activity in intact isolated KG-1a CD34^+^ cells using previously published methods [28]. Briefly, KG-1a CD34^+^ cells (5 × 10^5^) were incubated with 2 μM and 6 μM PepE or DMAPE (Previously reported TrxR1 inhibitor Parthenolide [29] was used as the positive control, 0.1% DMSO was used as the negative control) in 6- well plates for 12 h, then 10 μM TR-green was added to each plate and incubated at 37 °C for 3 h. Fluorescence and brightfield images were captured using an inverted fluorescence microscope IX-73 (Olympus) with Metamorph software (Molecular Devices).

### 2.18 Generation of TrxR1 knockdown and overexpression cells

For TrxR1 knockdown experiment, KG-1a CD34^+^ cells were seeded in a six-well plate at 2.5 × 10^5^/well in antibiotic-free IMDM medium for 24 h; then cells were washed once with 2 ml of shRNA transfection medium (Santa Cruz Biotech, sc-108062) and transfected with either TrxR1 shRNA plasmid (Santa Cruz Biotech, sc-36750-SH) or Control shRNA plasmid (negative control, Santa Cruz Biotech, sc-108060) according to the manufacturer's instructions. Briefly, for each transfection, 0.1 ml of shRNA (TrxR1 or control) duplex solution (containing 1 μg of shRNA plasmids) and 0.1 ml of transfection reagent (Santa Cruz Biotech, sc-108061) were gently mixed and incubated at room temperature for 30 min. The mixture was then diluted with 0.6 ml of shRNA transfection medium and overlay onto the washed cells. Cells were incubated at 37 °C in the 5% CO_2_ incubator for 7 h, then 1 ml of the IMDM medium containing 20% FBS were added, and incubated for another 24 h. All the medium were then removed and cells were suspended in IMDM containing 10% FBS, 100 U/mL penicillin and 100 μg/mL streptomycin medium for incubation and further cell viability assay. The knockdown efficiencies were evaluated by western blot and RT-qPCR analyses.

For TrxR1 overexpression experiment, KG-1a CD34^+^ cells were seeded in a six-well plate at 2.5 × 10^5^/well in antibiotic-free IMDM medium for 24 h, then cells were transfected with TrxR1 CRISPR activation plasmids (control CRISPR plasmids as a negative control) according to the manufacturer's instructions. Briefly, for each transfection, 0.15 ml of the Plasmid DNA solution (containing 3 μg of TrxR1 activation or control DNA plasmids) and 0.15 ml of diluted UltraCruz transfection reagent (Santa Cruz Biotech, sc-395739) were gently mixed and added to the cells. After incubation at 37 °C in the 5% CO_2_ incubator for 48 h, the medium was replaced by IMDM containing 10% FBS and 5 μg/ml of puromycin dihydrochloride medium for incubation and further cell viability assay. The activation efficiencies were evaluated by western blot and RT-qPCR analyses.

### 2.19 Trx1 redox state assays

The redox state of Trx1 was determined using Liu's method [30]. Phenylarsine oxide (PAO)-Sepharose was prepared by immobilization of PAO on Sepharose 4B according to Liu's report [30]. The reduced form was pulled down on the Sepharose, while the oxidized form remained in the solution. Briefly, the KG-1a CD34^+^ cells were treated with 5 μM PepE/DMAPE or vehicle control (DMSO) for 24 h. The cellular proteins were extracted by RIPA buffer and quantified by the Bradford method. The fully oxidized and reduced controls were prepared by incubating the non-treated cell lysate with diamide (2 mM) and TCEP (2 mM) for 1 h at 37 °C, respectively. The cell lysates were then incubated with PAO-sepharose on a rotating shaker for 1 h at room temperature. The supernatant was collected and the PAO-sepharose was washed with Tris-EDTA buffer. Then, the reduced Trx1 was eluted using Tris-EDTA buffer containing DPMS (10 mM). All samples were separated on SDS-PAGE and electrotransferred to PVDF membrane for western blot analyses. The bands were analyzed using Gel-pro 32 software.

### 2.20 Immunoprecipitation

The KG-1a CD34^+^ cells were treated with PepE or vehicle control (DMSO) for 12 h. The cellular proteins were extracted by RIPA buffer and quantified by the Bradford method. For each cell lysate, a volume containing 1 mg protein was mixed with 1 μg anti-Trx1 antibody coupled to 20 μL protein G-Sepharose (Abcam) and tumbled at 4 °C for 2 h. The Sepharose beads were then washed three times at 4 °C with a washing buffer containing 50 mM Tris (pH = 7.4), 0.5 M NaCl, and 1 mM EGTA. The immunoprecipitated proteins were eluted and denatured by 2×SDS-PAGE loading buffer and then boiled for 5 min. The eluted solutions were then centrifuged at 12000 g; the supernatants were collected and stored at −20 °C for SDS-PAGE and western blot analyses.

### 2.21 Statistical analysis

Data are presented as means ± SD of different determinations. Statistical differences between two groups were assessed by the Student's *t*-test. Comparisons among multiple groups were performed using one-way analysis of variance (ANOVA). A *P* value < 0.05 was used as the criterion for statistical significance.

## 3 Results

### 3.1 PepE and its amino derivative DMAPE eradicate primary AML progenitor cells in vitro

In our previous publication, we have examined the potency of PepE and its water-soluble amino-analog DMAPE against multiple cultivated AML cells [23]. The results showed that both PepE and DMAPE have potent activity against KG-1a CD34^+^ cells. We, therefore, selected these two promising compounds for further evaluation. In this study, we analyzed the effects of DMAPE and PepE treatment on primary APCs (CD34^+^ labeled) isolated from blood/bone marrow specimens of AML patients (AML1-5).

**Fig. 2:**
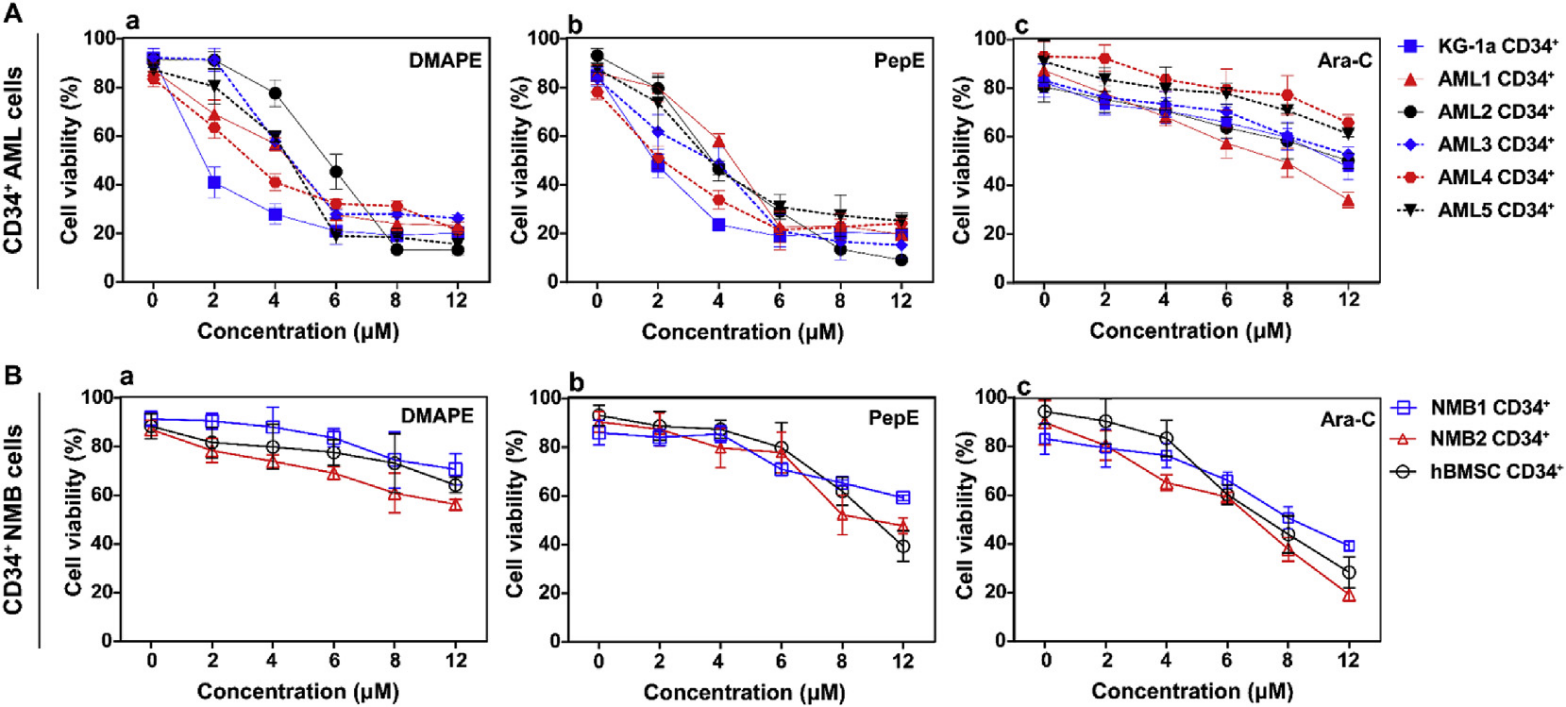
**PepE and DMAPE selectively against CD34**^**+**^**AML cells in a dose dependent manner.** In vitro cultures of were maintained for 48 h followed by analysis of cell viability using CCK-8. Each plot shows the average percent cell viability for CD34^+^ AML (A) and NBM cells (B) treated with different concentrations of DMAPE (A-a, B-a), PepE (A-b, B-b) or Ara-C (A-c, B-c). Each error bar represents the SD. All assays were performed in triplicate.

As shown in Fig. 2A-a&b, most primary CD34^+^ AML cells were very sensitive to both PepE and DMAPE treatment and show strong response starting at 6 μM (average 24.01% cells viable after PepE treatment and 28.85% cells after DMAPE treatment). On the other hand, at 6 μM concentration, both PepE and DMAPE had reduced toxicities on control CD34^+^ NBM cells and hBMSCs (Fig. 2B-a&b). Furthermore, when the drug concentration reached 12 μM, PepE started to exhibit modest effects on NBM and hBMSC cells, while DMAPE was relatively safe (Fig. 2B-a&b).

To further assess the relative efficacy of PepE and DMAPE, we performed a side-by-side comparison study with the standard anti-AML chemotherapy drug Ara-C on CD34^+^ AML or NBM (including hBMSCs) cells. As depicted in Fig. 2A-c&2B-c, Ara-C was more toxic than PepE and DMAPE on NBM and hBMSC cells while having no apparent toxicity against AML progenitor cells. This result is in agreement with an earlier report by Guzman et al., that Ara-C showed reduced toxicity on the AML progenitor population [18]. Taken together, these results indicated that both PepE and DMAPE exhibited strong and specific toxicity to Ara-C resistant primary CD34^+^ AML cells, while DMAPE showed less toxic effects than its parent drug PepE at high doses.

### 3.2 Selectively transformation from DMAPE to PepE in vitro

**Fig. 3:**
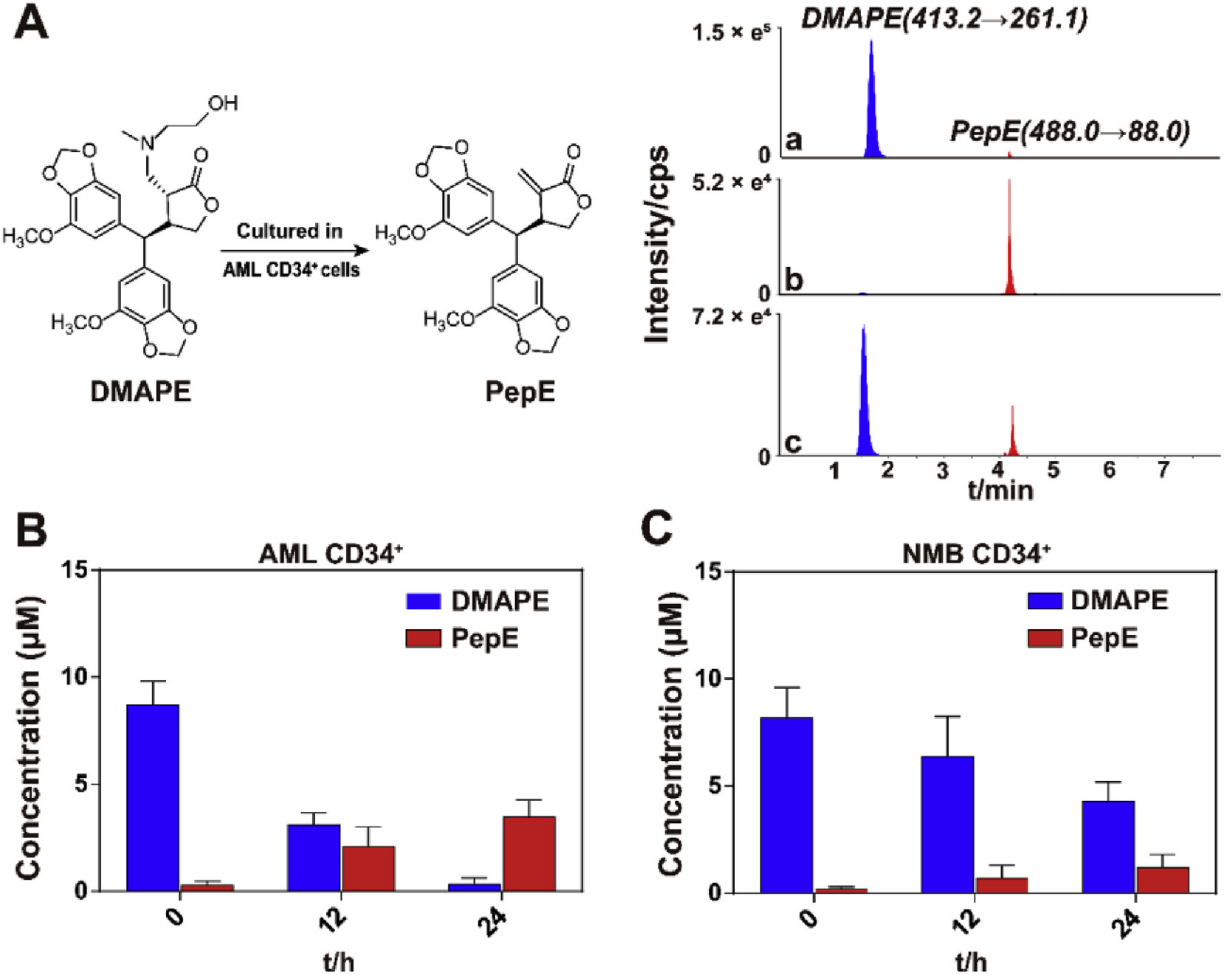
**Transformation from DMAPE to PepE in vitro.** (A) Representative UFLC-MS/MS multiple reaction monitoring chromatograms for DMAPE (blue) and PepE (red): (a) 10 μM of DMAPE cultured with cell culture medium without cells for 0 h; (b) 10 μM of DMAPE cultured with KG-1a CD34^+^ cells for 12 h; (c) 10 μM of DMAPE cultured with hBMSC cells for 24 h. (B) Concentrations of PepE and DMAPE from a 24 h time course starting with 10 μM of DMAPE in AML CD34^+^ cells (Data are presented as mean ± SD, n = 6). (C) Concentrations of PepE and DMAPE from a 24 h time course starting with 10 μM of DMAPE in NMB CD34^+^ cells (Data are presented as means ± SD, n = 3).

As we previously reported, DMAPE was stable in the cell cultural media (without cells) and no significant amount of PepE was detected within 24 h [23]. However, in the AML CD34^+^ cells group, PepE was released from DMAPE (Fig. 3A&B). The concentration ratio of PepE to DMAPE kept rising until DMAPE could not be detected after 24 h (Fig. 3A&B). In the NBM CD34^+^ (including hBMSCs) cells, on the other hand, the PepE release rate was significantly lower than that in the AML CD34^+^ cells; and about half of the DMAPE remained in the culture media after 24 h (Fig. 3C). Rather than DMAPE selectively releasing PepE when cultured with the APCs, this effect could be explained as APCs having special metabolic pathways to metabolize DMAPE and release PepE, while the NBM cells cannot metabolize DMAPE. It may be because of the presence of specific transporters on the cell membranes that transport DMAPE into the cells more efficiently in the APCs or because of the presence of special enzymes in the APCs that can break DMAPEs. The mechanism underlying the PepE release from DMAPE in APCs is being studied, which will report shortly.

### 3.3 Pharmacokinetic study of DMAPE *in vivo*

**Fig. 4:**
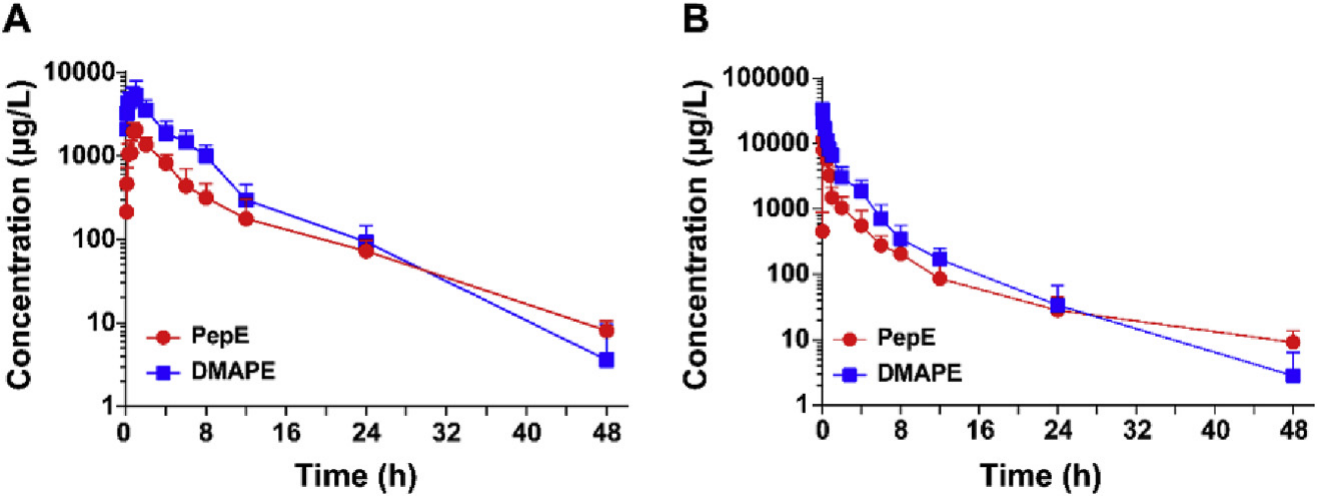
**Pharmacokinetic study of DMAPE in Rats.** DMAPE and PepE plasma concentration-time curve in rat after single i. g. administration of 50 mg/kg of DMAPE (A) or single i. v. administration of 50 mg/kg of DMAPE (B). Data are presented as means ± SD, n = 6.

To study the *in vivo* biotransformation of DMAPE, a UFLC-MS/MS method for simultaneous determination of DMAPE and PepE in rat plasma was developed and validated according to the US Food and Drug Administration for bioanalytical method validation protocol previously (data not shown). The mean plasma concentration-time curves of DMAPE and PepE obtained after oral administration of 50 mg/kg DMAPE are shown in Fig. 4A. The main pharmacokinetic parameters obtained using a non-compartmental model analysis are summarized in Table 1. The results indicated that the plasma concentrations of both DMAPE and PepE increased rapidly after the oral administration 50 mg/kg of DMAPE and both reached the peak concentration within 0.85 h. Then the concentrations of DMAPE and PepE declined, with the eliminated half-life (t_1/2_) values at 4.26 and 7.02 h, respectively. These data demonstrated that DMAPE not only can sustain the release of PepE *in vivo* but also can keep an effective PepE concentration range in the blood for a relatively long time. Next, the pharmacokinetics of DMAPE was studied by intravenous injection at the dose of 50 mg/kg. The corresponding plasma concentration-time curves and pharmacokinetic parameters are shown in Fig. 4B and Table 1. DMAPE has an oral bioavailability (F) of around 85%, which was much higher than that of PepE (around 14.1%, previously reported by our group) (Table 1) [31]. Taken together, the pharmacokinetic properties of DMAPE make it sufficiently promising as a drug candidate for further *in vivo* efficacy evaluation.

**Table 1:** Pharmacokinetic parameters of DMAPE and PepE after single administration of DMAPE aqueous solution at dose of 50 mg/kg (mean ± SD, n = 6).

Parameters	Intragastric admin (50 mg/kg)		Intravenous admin (50 mg/kg)	
	DMAPE	PepE	DMAPE	PepE
AUC _0-t_ (μg h/L)	25211.6 ± 8956.1	10635.2 ± 2926.4	29859.3 ± 7067.6	11305.8 ± 3520.3
AUC _0-∞_ (μg h/L)	25384.1 ± 8951.1	10698.1 ± 2927.3	29979.9 ± 7006.1	11417.2 ± 3517.4
C_max_ (μg/L)	5763.1 ± 2510.2	2298.1 ± 270.39	21653.3 ± 3594.1	8627.5 ± 2041.7
T_max_ (h)	0.83 ± 0.20	0.85 ± 0.13	–	0.21 ± 0.17
t_1/2_ (h)	4.26 ± 1.63	7.02 ± 1.30	4.33 ± 1.77	8.89 ± 4.80
MRT _0-t_ (h)	4.90 ± 0.42	7.32 ± 0.82	2.61 ± 0.86	3.91 ± 1.40
MRT _0-∞_ (h)	5.29 ± 0.32	7.75 ± 0.74	2.73 ± 0.85	4.64 ± 1.88
CL (L/h/kg)	2.14 ± 0.61	4.94 ± 1.22	1.74 ± 0.40	4.65 ± 1.10
Bioavailability, F	84.67%	93.70%	–	–

### 3.4 Efficacy of DMAPE in a murine model

**Fig. 5:**
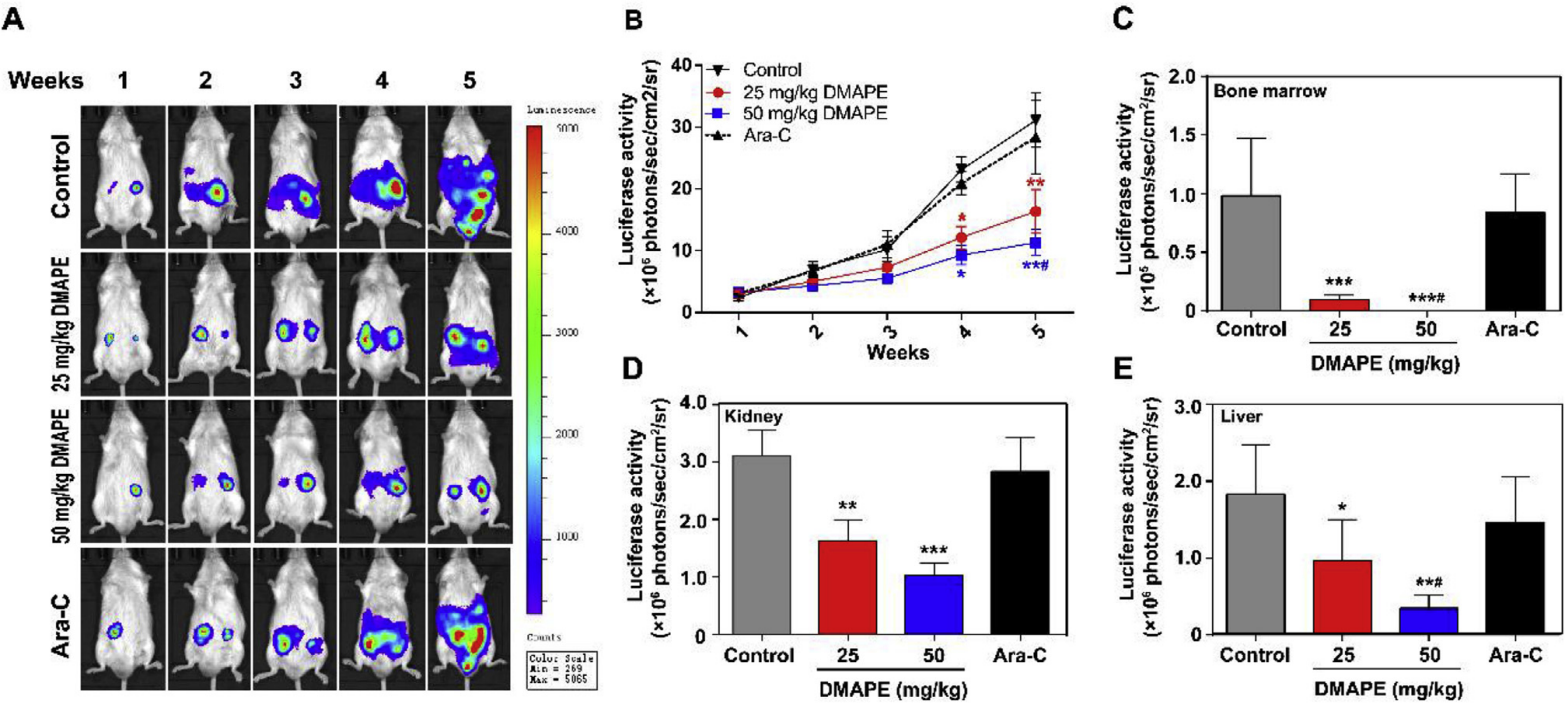
**Therapeutic efficacy of DMAPE on suppressing the growth of KG-1a CD34**^**+**^**cells in NOD-SCID mice model.** Enriched KG-1a CD34^+^ cells tagged with luciferase reporter gene were injected intravenously through vein into the NOD-SCID mice model. (A) Luciferase signal was followed at week 1–5 by injecting D-luciferin phosphate into the mice and visualized under bioluminescence imager. Mice were i. g. administrated with DMAPE (25 and 50 mg/kg), Ara-C (50 mg/kg) and Saline solution (negative control) daily. The color scale indicates the signal intensity, which is directly proportional to the number of KG-1a cells; (B) Quantitative analysis of the growth and metastatic of KG-1a cells by measurements of luciferase activity in mice treated with DMAPE (25 and 50 mg/kg), Ara-C and Saline solution at week 1–5, respectively. At week 5, bone marrows, kidneys and livers from both control and treated mice were excised and imaged, the bioluminescence signals of excised bone marrows (C), kidneys (D) and livers (E) were quantified for each group of mice. All above data are presented as means ± SD (n = 5). *P < 0.05, **P < 0.01, ***P < 0.001 when compared with control group. ^#^*P* < 0.05 when compared with 25 mg/kg DMAPE group.

As shown in Fig. 5A, the bioluminescence signal in the saline-treated mice accumulated mainly in the kidney and liver region; the signal continued to grow and invade to other organs, giving out a very strong signal five weeks after transplantation. In contrast, the DMAPE-treated groups showed no remarkable enhancement of luciferase signals. As shown in Fig. 5A&B, the signal intensities were significantly weaker when compared to the saline-treated mice (*P* < 0.05 at three weeks and *P* < 0.01 at five weeks of treatment). Moreover, the signal intensities of the 50 mg/kg DMAPE-treated mice were significantly weaker than that of the mice that received 25 mg/kg DMAPE at week five (*P* < 0.05). These indicated that the growth and metastasis of KG-1a CD34^+^ cells were inhibited by DMAPE in a dose-dependent manner *in vivo*. On the other hand, consistent with its in vitro activity, the *in vivo* efficacy of Ara-C against KG-1a CD34^+^ cells was not significant, and the mice treated with Ara-C showed strong luciferase signals after 5 weeks of treatment.

At the end of the experiment, all mice were euthanized, and their liver, kidney, and bone marrow were carefully excised and imaged. The average luciferase signal intensity in the bone marrow of saline-treated mice was 0.97 × 10^5^ photons/sec/cm^2^/sr, while in the 50 mg/kg DMAPE-treated almost no luciferase signal was detected, indicating a “KG-1a cell-free” state in the bone marrow of these animals (Fig. 5C). Furthermore, the overall luciferase signal in the liver and kidney, representing the extent of metastasized KG-1a cells in distant organs, was found significantly stronger in the saline-treated group than in the 50 mg/kg DMAPE-treated group (*P* < 0.001 in kidneys, *P* < 0.01 in livers, Fig. 5D&E). The above findings confirmed that, unlike the conventional chemotherapy drug Ara-C, DMAPE treatment was effective in inhibiting APCs-invasion to the bone marrow and other vital organs in the murine model, suggesting that it can be further developed as a new drug candidate for APCs-targeted therapy.

### 3.5 PepE and DMAPE effects on cell survival are mediated by changes in the oxidative state

**Fig. 6:**
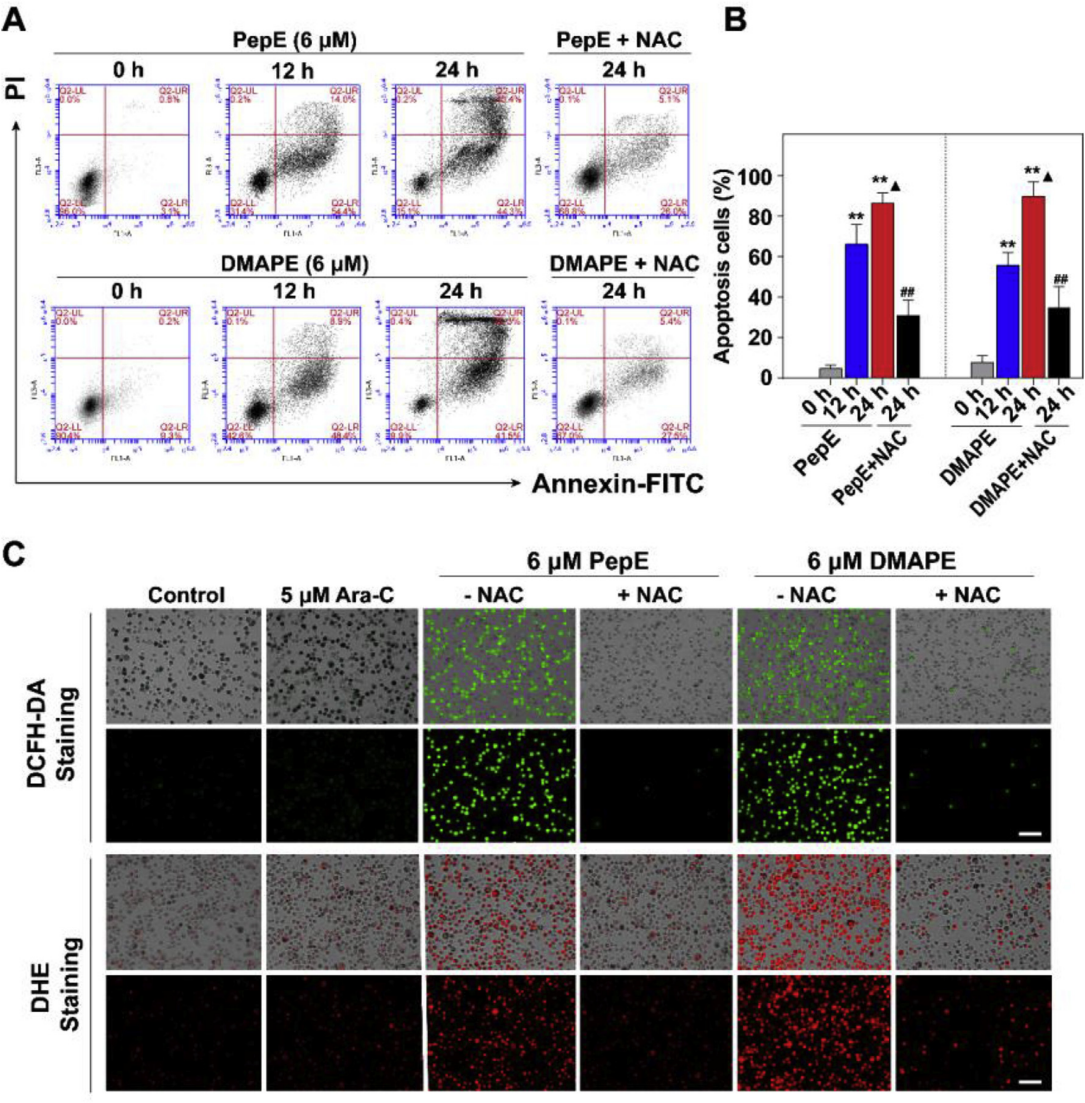
**Induction of apoptosis and ROS of CD34**^**+**^**AML cells by PepE and DMAPE.** (A) Analysis of apoptosis by the Annexin V/PI double staining assay. KG-1a CD34^+^ cells were treated with 6 μM of PepE or DMAPE for 12 h or 24 h, and representative FACS analysis scatter-grams for 10000 cells from 3 independent experiments were shown. The cells show four different cell populations designated as the follows: live cell population (lower left), early apoptosis population (lower right), and late apoptosis population (upper right) necrotic cell population (upper left). (B) The quantification of the apoptotic cells (early + late apoptosis) after the drug treatment (^∗∗^*P* < 0.01 when compared with 0 h control group, ^▲^*P* < 0.05 when compared with the 24 h group, ^##^*P* < 0.01 when compared with the non NAC-treated group). (C) Analysis of cellular ROS level by DCFA-DA or DHE staining. The bright field pictures (top panel) and the fluorescence pictures (bottom panel) were captured by inverted fluorescence microscope. *Scale bars:* 20 μm.

As shown in Fig. 6A&B, treatment of KG-1a CD34^+^ cells with 6 μM of PepE or DMAPE for 12 h caused ∼65% or ∼55% cell apoptosis, respectively. The apoptotic population increased to ∼85% or ∼90%, respectively when the cells were treated for 24 h. Furthermore, we also discovered that the apoptosis of triggered by PepE and DMAPE was completely inhibited by pretreatment of the cells with a high concentration of NAC (5 mM). NAC is a known small molecule antioxidant that neutralizes ROS, detoxifies xenobiotics, and regulates redox signaling in mammalian cells. Our observation that pretreatment of the KG-1a CD34^+^ cells with NAC totally impairs the cytotoxicity of PepE (or DMAPE) suggests that PepE and DMAPE induced cell death is mainly mediated through oxidative stress. The derived primary CD34^+^ AML cells shared the same results as the KG-1a cells (data not shown).

To further confirm this finding, we determined the ROS levels in KG-1a CD34^+^ cells after PepE (or DMAPE) treatment. DCFH-DA is a general ROS probe. DCFH-DA staining indicates the burst of ROS after stimulation of the cells with 6 μM of PepE (or DMAPE). Pretreatment of the cells with NAC blocks ROS accumulation (Fig. 6C). Furthermore, we employed another dye, DHE, to confirm ROS generation in the cells. DHE readily penetrates plasma membrane to intercept ROS, especially the superoxides, to form a product that intercalates with nucleic acids and emits red fluorescence. As expected, the PepE (or DMAPE) treated cells showed fluorescence, which was blocked by NAC pretreatment (Fig. 6C). Other primary AML CD34^+^ cells in consistence with the results of KG-1a CD34^+^ cells (data not shown). Taken together, these results suggest PepE (or DMAPE) induces apoptosis by promoting ROS accumulation in APCs.

### 3.6 Primitive AML progenitor cells differentially express genes/proteins required for the control of the oxidative state

**Fig. 7:**
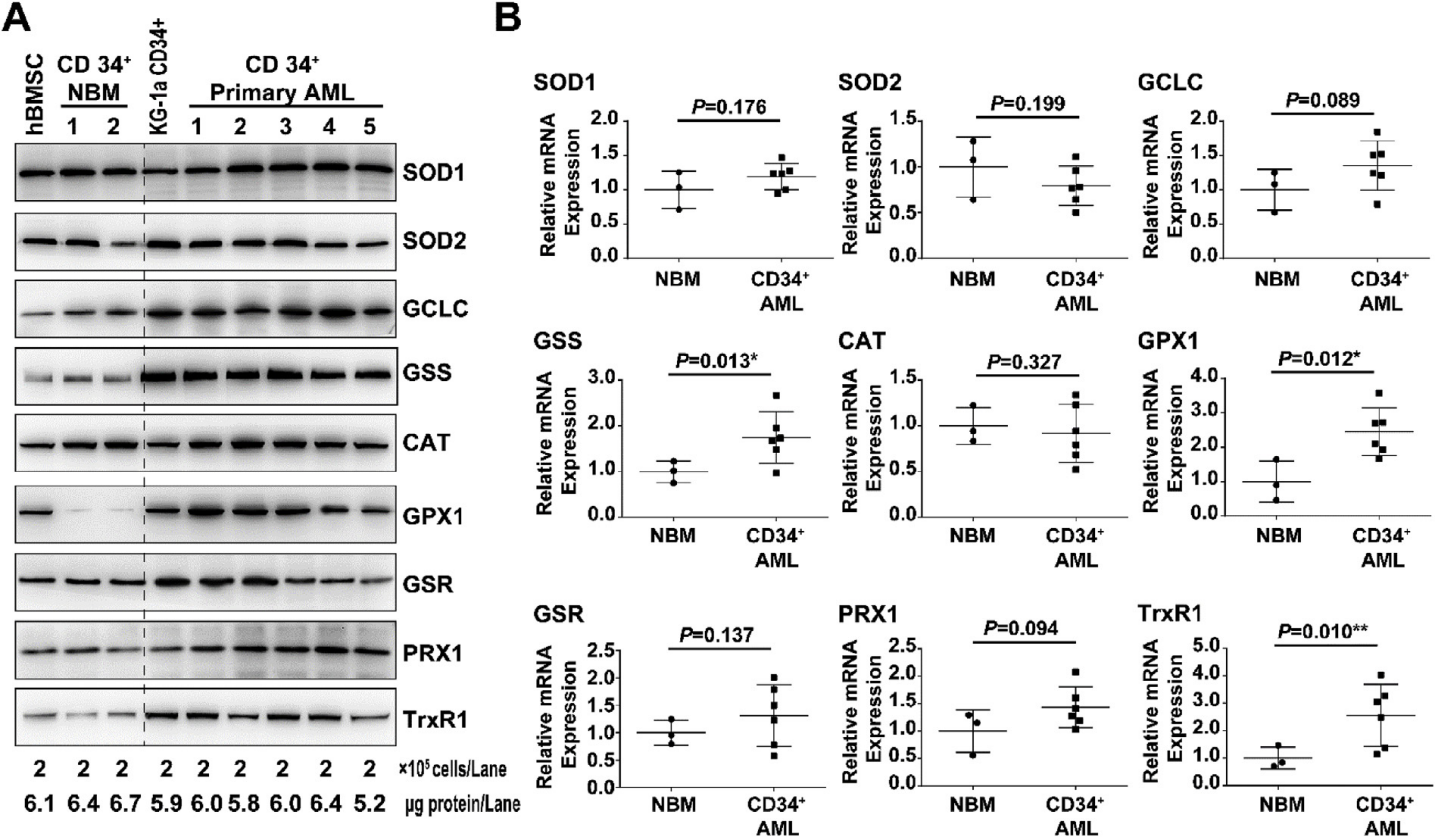
**CD34**^**+**^**AML cells differentially express genes required for control of redox state.** (A) Expression of major antioxidant proteins in human CD34^+^ NMB (n = 3) and CD34^+^ AML (n = 6) cells. (B) Relative mRNA expression of major antioxidant genes in human CD34^+^ NMB (n = 3) and CD34^+^ AML (n = 6) cells. Mean expression of HPRT1 was used as reference to internally normalize the expression of each gene within each cell lines. Average expression of each gene in CD34^+^ NMB (n = 3) cells was set to 1, and the relative expression of each gene in each cell lines was calculated accordingly and presented as square plot. Mean ± S.E. of each group is presented, ^∗^ and ^∗∗^ indicate significant differences.

To further investigate how PepE (or DMAPE) selectively affects the cellular redox state of APCs, we first compared the protein expression levels of all major antioxidant genes [32,33] between primary CD34^+^ AML and NBM (including hBMSCs) cells. It has been noted that the “house-keeping” genes like GAPDH in cancer tissues consistently differs from that of normal tissues [34]. Therefore, we chose to compare the protein expression of antioxidant genes from the same number of cells. As shown in Fig. 7A, probing the lysates from equal numbers of CD34^+^ AML (n = 6) and NBM (including hBMSCs) cells (n = 3) revealed comparable levels of SOD1, SOD2, CAT, and GSR proteins. However, GCLC, GSS, and GPX1 protein levels appeared to be significantly up-regulated in the majority of the primary CD34^+^ AML specimen, suggesting an aberrant glutathione metabolism in AML progenitor cells, which is in agreement with Pei's report [34]. Moreover, the TrxR1 protein, a key thioredoxin system enzyme known to maintain intracellular redox homeostasis and regulate multiple redox signaling [35], was also found overexpressed in our cohort of primary CD34^+^ AML specimens. TrxR1 is reported overexpressed in a number of human cancer cell lines and primary tumors, and its overexpression is associated with tumor aggressiveness, drug resistance, and poor prognosis [35]. This is the first time that TrxR1's overexpression was discovered in primary AML progenitor cells and specimens, which indicates a new molecular target for anti-APCs therapy.

To gain further insights into the differential expression of redox genes, we compared their mRNA levels. We used the mean expression of HPRT1 as a reference to internally normalize the expression of each gene within each specimen [34]. Average expression of each gene in CD34^+^ NBM (n = 3, including hBMSCs) cells was set to 1, and the relative expression of each gene in each specimen was calculated accordingly. As shown in Fig. 7B, mRNA levels of all the genes examined were consistent with the protein expression results. For example, CD34^+^ AML cells showed significantly up-regulated expression of GCLC (1.5 fold, *p* = 0.049), GSS (1.6 fold, *p* = 0.013), GPX1 (2.5 fold, *p* = 0.012), and TrxR1 (2.6 fold, *p* = 0.010) mRNA, consistent with the differences we observed in our western blot analyses. Taken together, these results demonstrated the up-regulation of glutathione and thioredoxin system components in CD34^+^ AML specimens.

### 3.7 PepE inhibits TrxR1 activity through covalent binding to its selenocysteine residue in C-terminal redox center

Reactive cysteine (Cys)/selenocysteine (Sec) sites are known to be important for the enzymatic function of the up-regulated differential proteins we discovered in AML CD34^+^ cells including GCLC, GSS, GPX1, and TrxR1 [[35], [36], [37], [38]]. Given the fact that PepE contains active (*α*-methylene-*γ*-lactone) moiety that may react with accessible Cys/Sec through Michael-type reaction, we deduced that PepE might inhibit the activities of one or more of the above-mentioned proteins by directly binding to them. To test this hypothesis, we employed the BLI assay to test the binding affinity between PepE and the differentially expressed proteins we discovered in AML specimens. As shown in Table S3, the affinity equilibrium constant revealed a high affinity between PepE and NADPH-reduced TrxR1, with K_D_ value observed at 0.34 μM. The binding affinity between PepE and the other three proteins, however, were much weaker than that with TrxR1, with K_D_ values of 534 μM for GPX1, 272 μM for GSS, and 1.34 mM for GCLC, respectively.

**Fig. 8:**
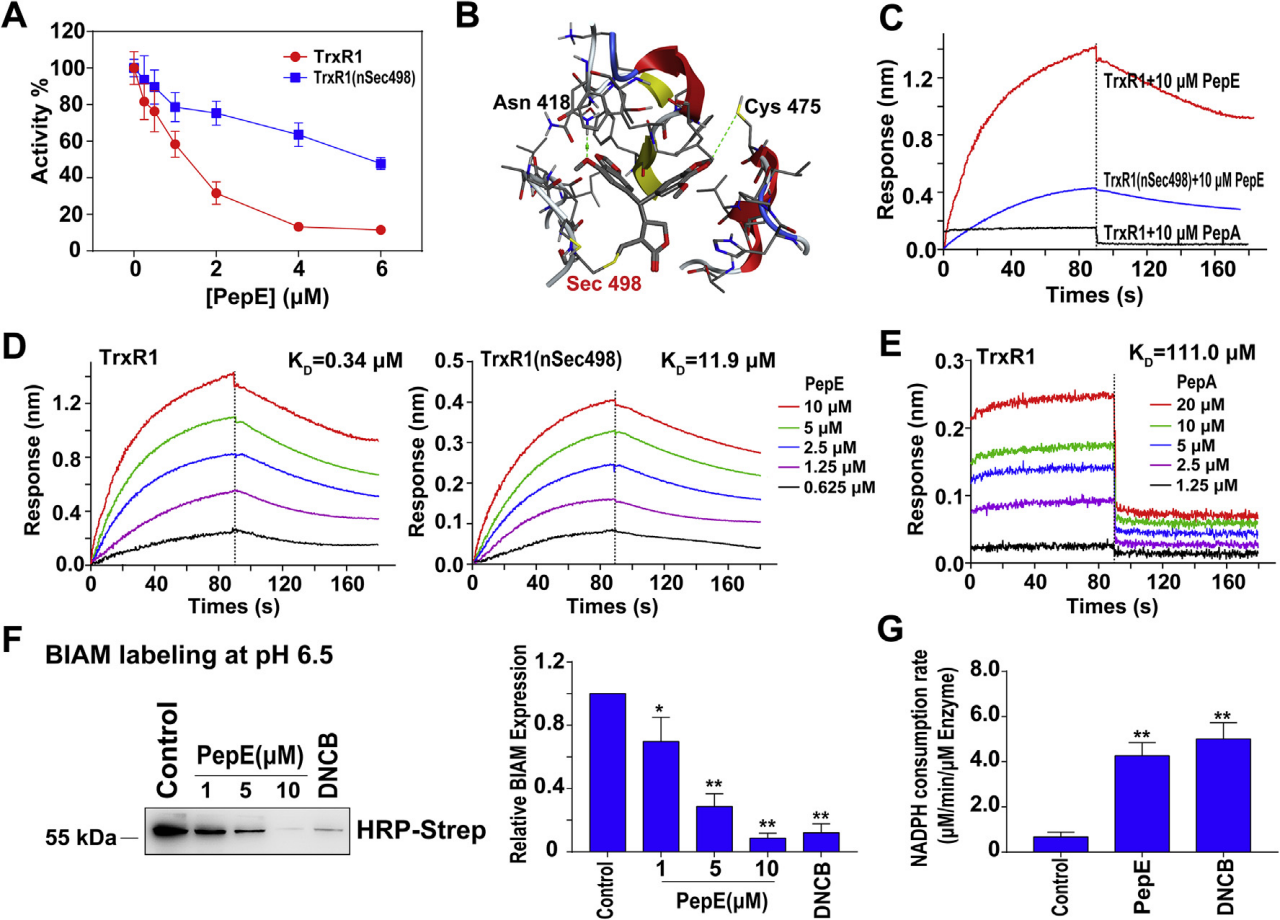
**PepE target the TrxR1 through binding its Sec residue.** (A) Inhibition assay of purified recombinant human TrxR1 or TrxR1 without Sec498 residue (nSec498, 0.1 μM) by PepE. The NADPH-reduced proteins were incubated with the indicated concentrations of PepE, and the enzyme's activity was determined by the DTNB assay (Data are presented as mean ± SD, n = 6). (B) Molecular docking of PepE with TrxR1 was carried out using the covalent docking protocol in the Schrodinger Suite. The yellow line indicated the covalent carbon bond, the green dotted lines indicate the hydrogen bonds. (C) Comparison of the binding activity between PepE and TrxR1 protein (red line), PepE and TrxR1 nSec498 protein (blue line), and PepA and TrxR1 protein (black line) through BLI analysis. (D) Kinetic analysis of the interaction between NADPH reduced TrxR1 (0.9 μM) or NADPH reduced TrxR1 nSec498 (0.9 μM) protein and PepE by BLI. The Super Streptavidin (SSA) biosensor tips coated with proteins (0.9 μM) were dipped in increasing concentrations of PepE (0.625, 1.25, 2.5, 5 and 10 μM) to measure the binding affinity of PepE to the proteins (K_on_) and subsequently moved to wells containing buffer to measure dissociation rates (K_dis_). The affinity constant (K_D_) was calculated as the ratio of the K_dis_ to the K_on._ (E) Kinetic analysis of the interaction between TrxR1 (0.9 μM) and different concentrations of PepA (1.25, 2.5, 5, 10 and 20 μM) by BLI. (F) The interaction of the PepE with the Sec residue in the *C*-terminal active center of TrxR1. HRP-conjugated streptavidin (HRP-Strep) detection of BIAM labeling of free Selenol in TrxR1 enzyme at pH 6.5 after this enzyme was incubated with different concentrations of PepE (1, 5 and 10 μM), DMSO(negative control), and DNCB (positive control, 5 μM), **P < 0.01, vs. the control group, data is presented as means ± SD in triplicate experiments. (G) The NADPH oxidase activity of TrxR1 (0.2 μM) induced by PepE (5 μM), DMSO (control), and DNCB (positive control, 5 μM). *P < 0.05 and **P < 0.01 vs. the control group, all data is presented as means ± SD, n = 6.

As PepE preferentially binds the TrxR1 enzyme, we next determined whether PepE inhibits TrxR1 using the DTNB reduction assay. As shown in Fig. 8A, incubation of PepE with the NADPH-reduced TrxR1 enzyme caused a dose-dependent inhibition of its activity with an IC_50_ value at 0.94 μM. This activity is more potent than that of the previously discovered Michael acceptor-based TrxR1 inhibitors such as parthenolide (approx. 3 μM) and shikonin (approx. 1.6 μM) [29,39]. Moreover, the removal of PepE from the incubation mixture by Sephadex G-25 desalting column did not recover the activity of this enzyme, suggesting that PepE inhibits TrxR1 irreversibly (data not shown).

TrxR1 is essential mammalian selenocysteine (Sec)-containing flavoenzymes with a –Gly-Cys-Sec-Gly- active site at *C*-terminal redox center. This very protein is the only enzyme catalyzing the NADPH-dependent reduction of the active site disulfide in Trxs, which plays essential roles in substrate reductions, defense against oxidative stress, and regulation by thiol redox control in cells [24,40]. TrxR1 has been reported overexpressed in many types of human cancer cells, thereby emerged as a valuable target for cancer treatment recently [35]. The Sec residue that determines the function of this enzyme (located in the easily accessible *C*-terminal redox center) is prone to be modified by the electrophilic agents such as PTL and curcumin. This modification then leads to the following oxidative stress-mediated apoptosis of cancer cells [35].

Since PepE contains an electrophilic moiety (α-methylene-γ-lactone), it is likely follows the same mechanism to bind to the Sec residue in TrxR1 to modify the enzyme. To conform this, we first calculated the binding mode for PepE and TrxR1 by using the covalent docking method. As shown in Fig. 8B, the carbon atom (yellow) indicated with an asterisk of PepE connects covalently with the selenium atom of Sec498. The binding pocket for PepE is formed by the C-terminal active site redox center of one subunit constituted by Gly496, Cys497, Sec498, and Gly499. Other than that, hydrogen bonds formed between PepE and the Cys475, Asn418 residues. These suggest that PepE may reacts with Sec498 of TrxR1 by forming a covalent bond and all the non-bonding interactions between PepE and residues around Sec498 seems to supply a favorable environment for the formation of the covalent bond.

Secondly, we compared the binding affinity and inhibition potency of PepE toward the Sec-containing TrxR1 enzyme (*C*-terminal peptide sequence as -SGASILQAGCUG-) and the Sec-missing enzyme (*C*-terminal peptide sequence as –SGASILQAGC-, containing the Cys497 but missing the Sec498 residue). As shown in Fig. 8A, C&D, PepE showed much weaker affinity and inhibition potency to the Sec-missing enzyme (K_D_ = 11.9 μM, IC_50_ > 8 μM) than the Sec-containing enzyme, which proved that Sec498 residue is crucial for PepE binding and inhibiting the TrxR1 enzyme. On the other hand, PepA, a PepE-analog which missing the electrophilic moiety (*α-methylene-γ-lactone*, Fig. S3) showed tiny affinity towards the enzyme (K_D_ = 111.0 μM Fig. 8E), indicated that the electrophilic moiety is extremely vital for PepE to target the enzyme.

Thirdly, BIAM labeling assay was used to further prove Sec498 residue is the main target for PepE modification [26,27]. In this assay, the reduced TrxR1 incubated with different concentrations of PepE (1, 5, 10 μM) or positive control DNCB (5 μM) was next probed with BIAM at pH 6.5, which allows preferential alkylation of Sec residues but not Cys residues due to the difference in pKa between Sec and Cys [27]. As shown in Fig. 8F, treatment with PepE or DNCB significantly block the BIAM alkylation of Sec residue in a dose dependent manner, which confirmed that Sec residue in the enzyme was the target for PepE modification.

The chemical modification of the *C*-terminal Sec residue of TrxR1 yields a SecTRAP (selenium compromised thioredoxin reductase-derived apoptotic protein). The SecTRAP lose the ability to reduce oxidized Trx1, but maintain NADPH oxidase activity in constantly generating ROS, which eventually contributes to increased intracellular oxidative stress and promotes both apoptosis and necrosis in cells [41]. Finally, we examined the NADPH oxidase activity by measuring the NADPH consumption rate to confirm whether SecTRAP was formed after treatment of PepE. As shown in Fig. 8G, when incubated with 5 μM PepE, the consumption of NADPH by TrxR1 was increased nearly 7-fold relative to the control and was comparable to that of DNCB (5 μM), which was used as a positive control in the experiment. These indeed suggest the formation of SecTRAP after PepE treatment.

### 3.8 PepE and DMAPE selectively inhibits TrxR1 activity in APCs

**Fig. 9:**
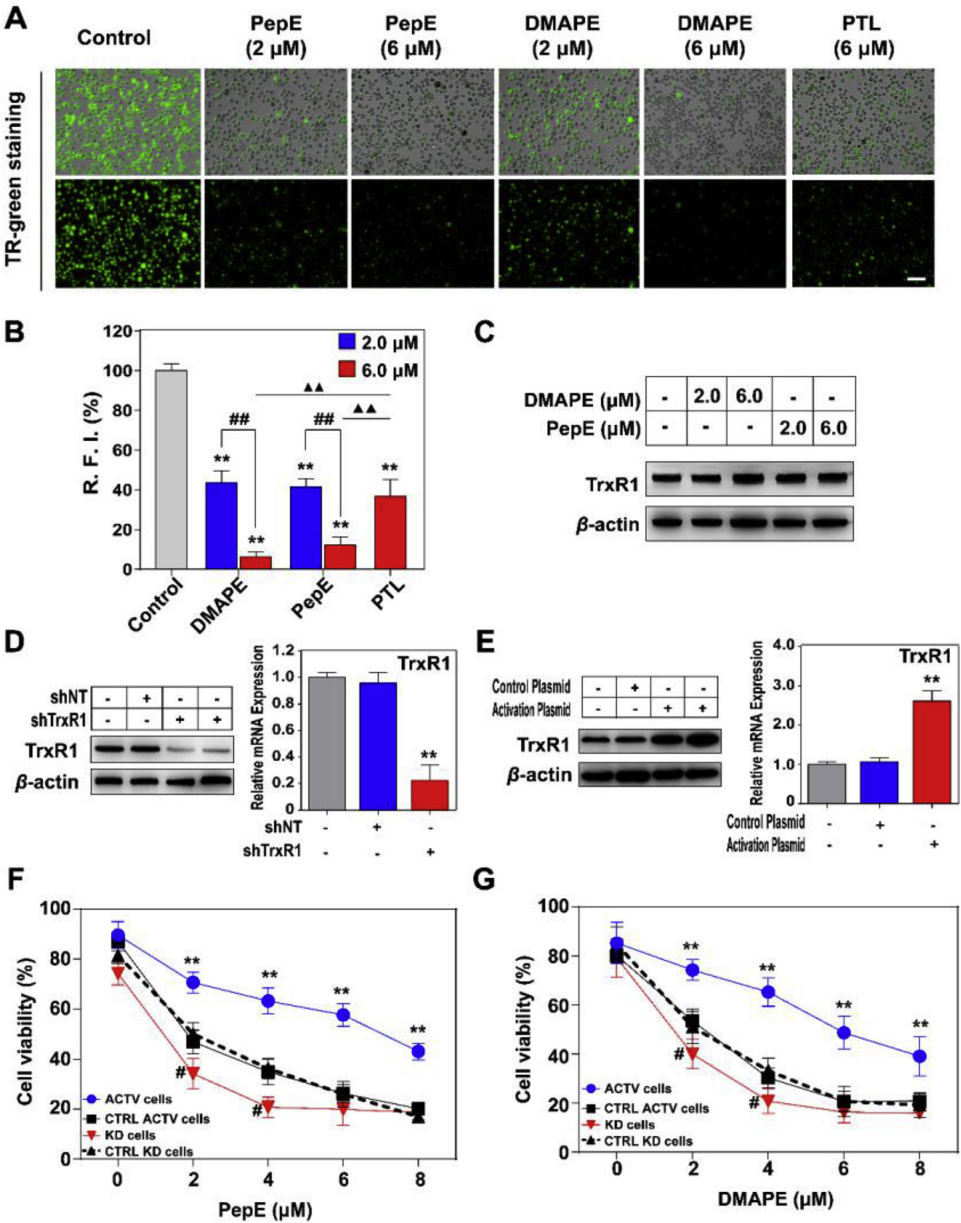
**PepE and DMAPE targeting the TrxR in CD34**^**+**^**AML cells.** (A) Imaging the TrxR activity in live KG-1a CD34^+^ cells by TR-Green. After treatment of cells with 2 and 6 μM of PepE, DMAPE or 6 μM of PTL (positive control) for 24 h, the TrxR activity was stained by the TrxR probe TR-Green. The bright field pictures (top panel) and the fluorescence pictures (bottom panel) were captured by inverted fluorescence microscope. *Scale bar:* 20 μm. (B) 10 cells were randomly selected and relative fluorescence intensity (RFI) was quantified in individual cells Metamorph Software (^∗∗^*P* < 0.01 vs. the control group; ^##^*P* < 0.01 vs. the 2.0 μM group, ^▲▲^*P* < 0.01 vs. the PTL group). Data are expressed as means ± SD of three experiments. (C) No obvious changes of TrxR1 expression levels in KG-1a CD34^+^ cells after PepE and DMAPE treatment. Cells were treated with the indicated concentration of DMAPE and PepE for 24 h, and the cell extracts were assessed by Western blots. (D) Quantification of TrxR1 expressions in control and *KG-1a TrxR1 knockdown* cells. TrxR1 expressions in different cells were analyzed by western blot using *β*-actin as the internal standard and qRT-PCR using GAPDH as the internal standard (means ± SD, n = 3, ^∗∗^*P* < 0.01 vs. the control cells). (E) Quantification of TrxR1 expressions in control and *KG-1a TrxR1 activation cells*. TrxR1 expressions in different cells were analyzed by western blot using *β*-actin as the internal standard and qRT-PCR using GAPDH as the internal standard. (means ± SD, n = 3, ^∗∗^*P* < 0.01 vs. the *control activation cells*). (F) and (G) Growth inhibition of PepE and DMAPE to *TrxR1 knockdown cells (KD cells)*, *control knockdown cells (CTRL KD cells)*, *TrxR1 activation cells (ACTV cells)* and *control activation cells (CTRL ACTV cells)*. These cells were treated with PepE or DMAPE for 48 h, and the viability was determined by the CCK-8 method. Data were expressed as mean ± SD of three experiments. ^∗^*P* < 0.05 and ^∗∗^*P* < 0.01 vs. the *CTRL ACTV cells*, ^##^*P* < 0.01 vs. the *CTRL KD cells*.

To broaden the understanding, we employed TR-green, a specific TrxR1 fluorescence probe reported by Huang et al. [25], to assess cellular TrxR1 activity. As shown in Fig. 9A&B, treatment of KG-1a CD34^+^ cells with PepE (or DMAPE) can cause a significant decrease in the fluorescence intensities in a dose-dependent manner, demonstrating the inhibition of TrxR1 by PepE (or DMAPE). It was also illustrated in Fig. 9A&B that the inhibition potency of PepE or DMAPE was significantly higher than PTL, a previously reported Michael acceptor-based TrxR1 inhibitor [29]. Moreover, as illustrated by Fig. 9C, there appears to be no apparent change in TrxR1 expression after the KG-1a CD34^+^ cells were treated with PepE or DMAPE for 24 h, indicating that the decreased enzyme activity we observed is caused by the direct inhibition of TrxR1.

To further verify the biological relevance of TrxR1-mediated cytotoxicity of PepE and DMAPE, we determined the cytotoxicity of PepE and DMAPE after knocking down TrxR1 expression by TrxR1 shRNA plasmid in KG-1a CD34^+^ cells (KD cells). Knockdown of TrxR1 in KG-1a cells were assessed through protein and gene expression experiments (Fig. 9D). As illustrated in Fig. 9F&G, PepE and DMAPE displayed elevated potency toward TrxR1 KD cells compared to the cells transfected with the negative control plasmid (CTRL KD cells). To further demonstrate whether the inhibition of TrxR1 is involved in the cytotoxicity of PepE, we compared the sensitivity of the KG-1a cells that were stably transfected with TrxR1 CRISPR activation (or negative control) plasmids toward PepE (or DMAPE) treatment. The transfection efficiency was determined by measuring the enzyme expression through western-blot and RT-qPCR analysis (Fig. 9E). As depicted in Fig. 9F&G, PepE and DMAPE both showed significant lower toxicity against TrxR1 overexpressed KG-1a cells (ACTV cells) than the control cells (CTRL ACTV cells). Taken together, these results indicate that TrxR1 is highly involved in the cellular actions of both PepE and DMAPE against APCs.

### 3.9 PepE and DMAPE inhibits TrxR1 activity lead to Trx1 oxidation and induction of ASK1-mediated apoptotic cell death

TrxR1 plays a critical role in the catalysis of the NADPH-dependent reduction of disulfide in the oxidized Trx to generate the reduced Trx, which interacts with multiple downstream proteins via a general thiol-disulfide exchange reaction [35]. Therefore, we determined the ratio of reduced Trx to oxidized Trx in KG-1a CD34^+^ cells after PepE or DMAPE treatment.

**Fig. 10:**
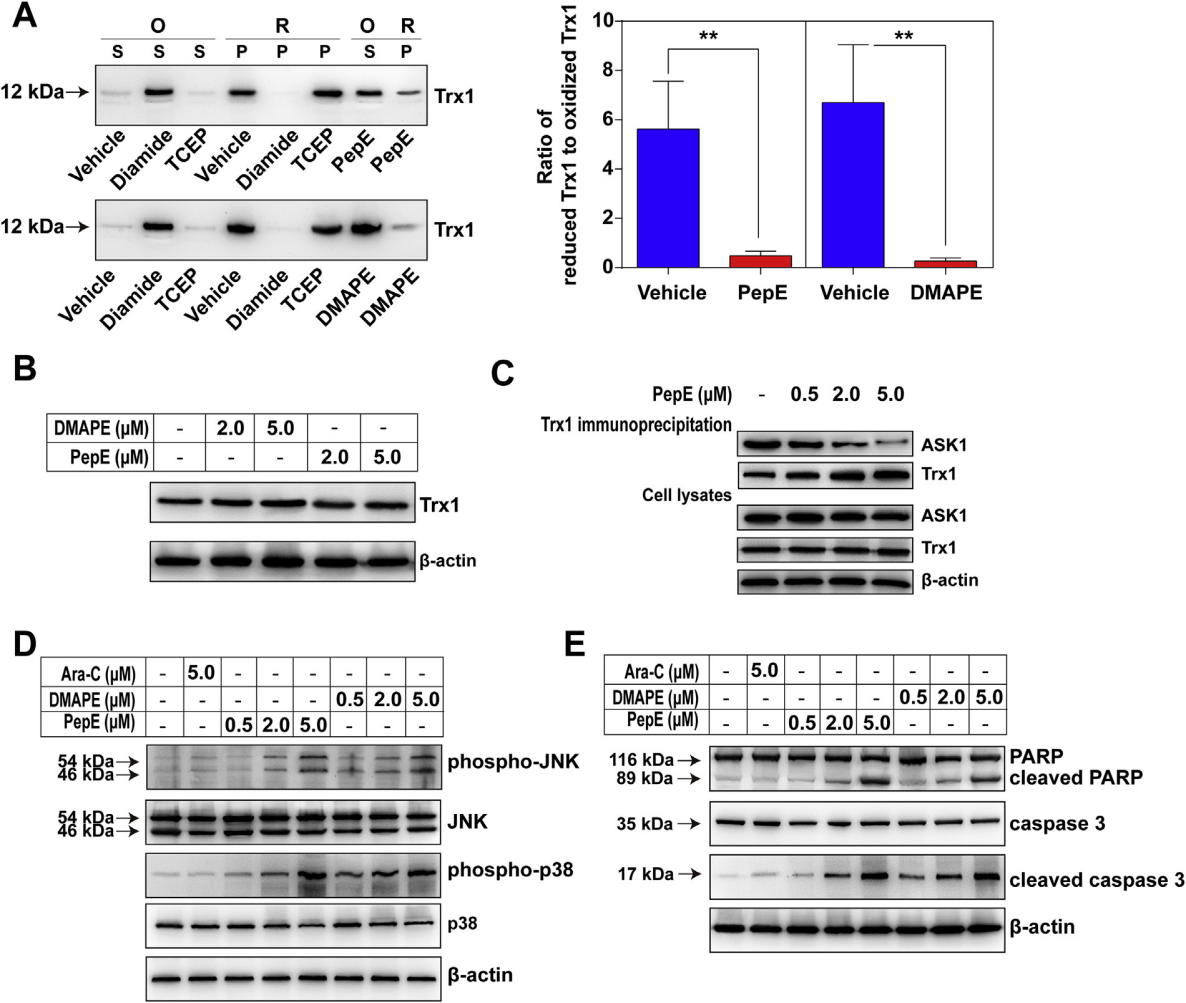
**Effects of PepE and DMAPE on Trx1 oxidation, Trx-ASK1 interaction and related apoptosis induction pathways in KG-1a CD34**^**+**^**cells.** (A) Determination of the oxidized Trx1 by the pull down assay. The Diamide- and TCEP-treated cell lysates were run to confirm the assay works. KG-1a CD34^+^ cells were treated with PepE and DMAPE (5 μM) for 24 h, and the Trx1 redox states were measured. S: samples in the supernatant; P: samples eluted from the PAO-sepharose; O: oxidized form; R: reduced form. Quantification of the ratio of the reduced Trx1 to the oxidized Trx1 by measuring the band density with Gel-pro 32. Data were expressed as mean ± SD of three experiments. ^∗∗^*P* < 0.01 vs. the vehicle groups. (B) No apparent alteration of the Trx1 expression after PepE and DMAPE treatment. KG-1a CD34^+^ cells were treated with 2 and 5 μM of PepE or DMAPE for 24 h, and protein level of Trx1 was illustrated by western-blots. (C) Effects of PepE and DMAPE on Trx-ASK1 interaction. Lysates from KG-1a CD34^+^ cells after 4 h treatment of PepE were subjected to immunoprecipitation using anti-Trx1 antibody. Immuno-precipitates and aliquots of the cell lysates were subjected to the SDS-PAGE and western-blot analysis using the indicated antibodies. (D) Dose-dependent effects of PepE and DMAPE on MAPK activation. Lysates of cells after 12 h of treatment with PepE, DMAPE, Ara-C or DMSO were collected and the protein levels of phosphorylated and total JNK and p38 were analyzed by western blot. (E) Dose-dependent induction of apoptosis, indicated by presence of cleaved caspase 3 and PARP in KG-1a CD34^+^ cells treated with PepE or DMAPE for 12 h. All Western blot images shown are representative of three independent experiments.

Following Liu's report, PAO-Sepharose was prepared to pull down the reduced Trx and leave the oxidized Trx in the solution. The reduced Trx was then released from the PAO-sepharose by the addition of DMPS. To validate this method, we treated the cell lysates with diamide or TCEP to generate the fully oxidized Trx or reduced Trx, respectively [30]. As shown in Fig. 10A, Trx from the diamide-treated lysate (oxidized form, O) is only present in the supernatant (S), whereas that from the TCEP-treated lysate (reduced Trx, R) is fully captured by the PAO-sepharose (P), demonstrating this method is suitable to distinguish the reduced Trx from the oxidized one. After validation, we adopted this method to determine the redox state of Trx. As shown in Fig. 10A, the majority of Trx was found in the DMSO (vehicle)-treated cells. The oxidized form was significantly increased and became more abundant than the reduced form after the treatment of KG-1a CD34^+^ cells with 5 μM PepE or DMAPE. The reduced Trx and oxidized Trx were quantified by measuring the band intensity, the ratio of the reduced form to the oxidized form is illustrated in Fig. 10A. As shown in Fig. 10B, there is no apparent alteration in the total Trx expression after PepE and DMAPE treatment.

Trx exerts anti-apoptotic effects by the suppression of ASK1-dependent apoptosis through a direct inhibitory protein-protein interaction between reduced Trx and ASK1. This protein-protein interaction is dependent on the redox state of Trx; when the Trx protein is in oxidized form, the Trx-ASK1 complex will dissociate and the downstream apoptotic pathway will be triggered [42,43]. Therefore, we next investigated the Trx and ASK1 interaction in KG-1a CD34^+^ cells after PepE treatment. As illustrated in Fig. 10C, Trx immunoprecipitation from lysates of KG-1a CD34^+^ cells treated with different concentrations (0.5, 2.0, 5.0 μM) of PepE revealed a dose-dependent decrease in the amount of ASK1 bound to Trx1. The dissociation between Trx1 and ASK1 protein in KG-1a cells then activated the ASK1 downstream MAPKs pathway, including the phosphorylation of p38 and JNK in a dose-dependent manner (Fig. 10D). The ASK1 dependent activation of p38 and JNK MAPK pathways finally triggered the apoptotic death of AML cells, as evident from a dose-dependent elevation of apoptotic markers such as cleaved PARP and caspase 3 (Fig. 10E). Taken together, these results indicated that the inhibition of TrxR1 by PepE (or DMAPE) led to the cellular oxidation of Trx, increased cellular oxidative stress, activated ASK1, activating the MAPKs pathways, which finally led to the apoptotic death of the AML progenitor cells.

## 4 Discussion

Conventional chemotherapies for AML affect only the differentiated or differentiating malignant cells. However, in many cases, a small population of immature APCs could remain untouched and subsequently cause the relapse of AML [9]. Therefore, APCs are now becoming a major target for clinical AML treatment. Unfortunately, to date, only a few agents have been reported to target the APCs, and most of them are still in the early stages of development [11]. In this study, a newly discovered natural compound-PepE was found to induce apoptosis of human primary CD34^+^ AML cells in the 4–6 μM range with almost no toxicity on the normal hematopoietic cells. By comparison, Ara-C (a conventional AML chemotherapy drug) at the same concentration range showed no apparent effect on AML CD34^+^ cells but had relatively high toxicity toward normal cells. DMAPE, a previously reported water-soluble amino-analog of PepE, was evaluated for its pharmacologic effect against primary CD34^+^ AML cells in this study. DMAPE not only retains the activity but also increases the safety by releasing its parent drug – PepE selectively in AML CD34^+^ cells. Further preliminary pharmacokinetic (PK) study revealed that this water-soluble analog had significantly higher oral bioavailability (85%) than that of PepE (14%), making it an excellent candidate for *in vivo* studies. Animal experiments confirmed its *in vivo* efficacy and the treatment with DMAPE at 25 mg/kg/d dose significantly suppressed the growth and metastasis of AML CD34^+^ cells in NOD/SCID mice. The *in vivo* efficacy of Ara-C, on the other hand, was not obvious. Together, the *in vitro* and *in vivo* data suggests that DMAPE could serve as a novel anti-APCs drug candidate for further clinical investigations.

The CD34^+^ AML progenitor cells, unlike the other AML cells, were found to have very low levels of ROS. It was reported that these cells are more sensitive to increases in ROS levels than the normal bone marrow cells. Therefore, increased ROS production or inhibition of the cellular antioxidant systems is increasingly considered as a therapeutic strategy to target the APCs [12,14]. In this study, we discovered that pretreatment of KG-1a CD34^+^ cells with the free radical scavenger NAC completely abrogated the DMAPE (or PepE)-mediated apoptosis, indicating that the apoptosis-inducing ability of DMAPE (or PepE) is attributed to its capability of promoting ROS production in APCs. To further investigate how DMAPE (or PepE) manipulates the ROS levels and eliminates the APCs, we first compared the crucial regulators of ROS signaling in primitive CD34^+^ AML patients specimens versus normal specimens. In line with the previously published studies, our data indicated that CD34^+^ AML cells have significantly elevated expression of glutathione pathway regulatory proteins including GCLC, GPX1, and GSS [34]. Most importantly, a key enzyme from thioredoxin systems – TrxR1, was discovered to be significantly overexpressed in primary CD34^+^ AML cells in this study. Subsequent BLI assay results indicated that among the four aberrantly expressed proteins we discovered in APCs, PepE preferably binds with TrxR1, with a K_D_ value of 0.34 μM.

Human TrxR1 contains a selenol side chain (from the Sec residue) at its *C*-terminal which determines the basic functions of TrxR1. Sec, compared with Cys, is more reactive and prone to be modified by various electrophiles [35]. Thus, we hypothesized that the *α*-methylene-*γ*-lactone (Michael acceptor) moiety of PepE covalently binds to the Sec moiety within TrxR1, thereby modifying its functions. DTNB reduction assay proved that PepE can irreversibly inhibit TrxR1 activity, with an IC_50_ value of 0.94 μM. The missing of Sec residue sharply decreased the sensitivity of the enzyme to PepE. The selective inhibition of Sec-containing TrxR1 but not Sec-missing TrxR1 proves a specific interaction between PepE and the Sec residue of the TrxR1. The following BIAM labelling and NADPH oxidase activity assay have further confirmed the facts that the Sec residue of the enzyme was modified and followed by the formation of SecTRAP with production of ROS after the treatment of PepE.

Further evidence in the cellular context to support the unique role of TrxR1 and the anti-APCs action of PepE (or DMAPE's) was also provided. KG-1a CD34^+^ cells stably overexpressing TrxR1 show less growth inhibition compared to cells transfected with control plasmids after PepE (or DMAPE) treatment. Moreover, the knockdown of TrxR1 elevated PepE (or DMAPE's) cytotoxicity, which further proves that TrxR1 is critically involved in the biological function of PepE (or DMAPE) against APCs. As a result of TrxR1 suppression, oxidized Trx was significantly accumulated in the cells. The dissociation of the ASK1-Trx complex and the activation of the p38 and JNK signal pathways in KG-1a CD34^+^ cells upon treatment with PepE (or DMAPE) were then observed by our tests.

**Fig. 11:**
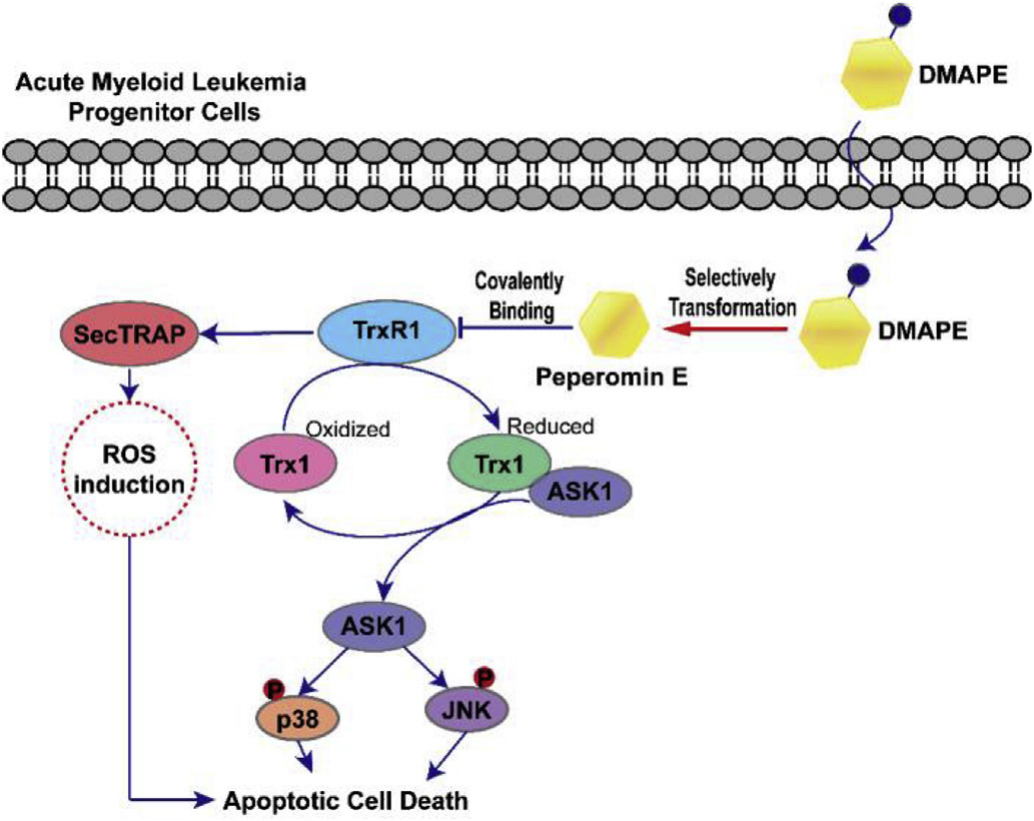
The proposed signal pathway for DMAPE-induced oxidative stress-mediated apoptosis of APCs.

In summary, as depicted in Fig. 11, we proposed that the ROS accumulation and apoptotic cell death of APCs upon PepE (DMAPE)-mediated TrxR1 inhibition can be triggered via following pathways. Firstly, inhibition of TrxR1 by PepE can resulted in formation of SecTRAP, thus converting the TrxR1 into a pro-oxidant NADPH oxidase. The SecTRAP, as reported previously, can resulted in rapid induction of ROS which finally leads to apoptosis of mammalian cells [41]. Secondly, inhibition of TrxR1 resulted in increased levels of oxidized Trx, therefore prevent Trx of providing reducing equivalents to peroxiredoxins, which will further aggravate the oxidative stress and cell apoptosis as a result of impaired antioxidant defense systems [35]. Finally, the increased oxidation level of Trx triggered by TrxR1 inhibition leading to dissociation of Trx-ASK1 complex, followed by activation of ASK1 and its downstream JNK and p38 MAPK signaling pathways, which finally causing the apoptotic death of cells.

The TrxR1 has emerged as an important target in cancer chemo-therapy, due to its overexpression in a variety of human cancer types (i.e. breast, gastric) and association with increased tumor growth, drug resistance and poor patient prognosis. Increasing numbers of TrxR1 inhibitors including metal-containing molecules (i.e. Auranofin, Cisplatin), selenium-containing molecules (i.e.1,2-[bis(1,2-benzisoselenazolone-3(2H)-ketone)] ethane), and natural derived chemicals (i.e. Parthenolide, Manumycin A, Curcumin, Juglone, and Piperlongumine) [28,29,44,45] have been reported as leads for the anti-cancer treatment in past few years. In this study, the observation of TrxR1 overexpression in clinical CD34^+^ AML samples sheds new light on the possibility of treating AML (especially relapsed AML) patients with TrxR1 inhibitors. In addition, the discovery of the strong inhibition ability of PepE against TrxR1 provides a novel TrxR1 inhibitor template for further studies. This very agent and its bioavailable analog may test clinically not only for eradicating APCs but also for treating variety of other TrxR1-overexpressing human cancers in near future.

## Ethics approval

Mice were maintained in specific-pathogen free (SPF) conditions in Nanjing University of Chinese Medicine. All animal studies were conducted under the guidelines approved by the Animal Care and Use Committee of the Nanjing University of Chinese Medicine (Nanjing, China).

## Declaration of interests

None.

## References

[1] Dohner H., Weisdorf D.J., Bloomfield C.D. (2015). Acute myeloid leukemia. N. Engl. J. Med..

[2] Tallman M.S., Gilliland G.D., Rowe J.M. (2005). Drug therapy for acute myeloid leukemia. Blood.

[3] Roboz G.J. (2012). Current treatment of acute myeloid leukemia. Curr. Opin. Oncol..

[4] Dombret H., Gardin C. (2016). An update of current treatments for adult acute myeloid leukemia. Blood.

[5] Bonnet D., Dick J.E. (1997). Human acute myeloid leukemia is organized as a hierarchy that originates from a primitive hematopoietic cell. Nat. Med..

[6] Mao M., Fu G., Wu J.S., Zhang Q.H., Zhou J., Kan L.X., Huang Q.H., He L.B., Gu B.W., Han Z.G., Shen Y., Gu J., Yu Y.P., Xu S.H., Wang Y.X., Chen S.J., Zhu Z. (1998). Identification of genes expressed in human CD34^+^ hematopoietic stem/progenitor cells by expressed sequence tags and efficient full-length cDNA cloning. P. Natl. Acad. Sci..

[7] Costello R.T., Mallet F., Gaugler B., Sanity D., Arnoulet C., Gastaut J.A., Olive D. (2000). Human Acute myeloid leukemia CD34^+^/CD38^-^ progenitor cells have decreased sensitivity to chemotherapy and Fas-induced apoptosis, reduced immunogenicity, and impaired dendritic cell transformation capacities. Cancer Res..

[8] Guan Y., Gerhard B., Hogge D.E. (2003). Detection, isolation, and simulation of quiescent primitive leukemic progenitor cells from patients with acute myeloid leukemia. Blood.

[9] Shlush L.I., Mitchell A., Heisler L., Abelson S., Ng S.W.K., Trotman-Grant A., Medeiros J.J.F., Rao-Bhatia A., Jaciw-Zurakowsky I., Marke R., Mcleod J.L., Doedens M., Bader G., Voisin V., Xu C.J., McPherson J.D., Hudson T.J., Wang J.C.Y., Minden M.D., Dick J.E. (2017). Tracing the origins of relapse in acute myeloid leukaemia to stem cells. Nature.

[10] Dick J.E., Lapidot T. (2005). Biology of normal and acute myeloid leukemia stem cells. Int. J. Hematol..

[11] Pollyea D.A., Jordan C.T. (2017). Therapeutic targeting of acute myeloid leukemia stem cells. Blood.

[12] Pollyea D.A., Gutman J.A., Gore L., Smith C.A., Jordan C.T. (2013). Targeting acute myeloid leukemia stem cells: a review and principles for the development of clinical trials. Haematoloigica.

[13] Guzman M.L., Neering S.J., Upchurch D., Grimes B., Howard D.S., Rizzieri D.A., Luger S.M., Jordan C.T. (2001). Nuclear factor-kB is constitutively activated in primitive human acute myelogenous leukemia cells. Blood.

[14] Samimi A., Kalantari H., Lorestani M.Z., Shirzad R., Saki N. (2018). Oxidative stress in normal hematopoietic stem cells and leukemia. J. Pathol. Microbiol. Immunol..

[15] Lagadinou E.D., Sach A., Callahan K., Rossi R.M., Neering S.J., Minhajuddin M., Ashton J.M., Pei S.S., Grose V., O'Dwyer K.M., Liesveld J.L., Brookes P.S., Becker M.W., Jordan C.T. (2013). BCL-2 inhibition targets oxidative phosphorylation and selectively eradicates quiescent human leukemia stem cells. Cell Stem Cell.

[16] Wang Y.Z., Krivtsov A.V., Sinha A.U., North T.E., Goessling W., Feng Z.H., Zon L.I., Armstrong S.A. (2010). The Wnt/β-Catenin pathway is required for the development of leukemia stem cells in AML. Science.

[17] Zhao M., Chen A., Jamieson C.H., Fereshteh M., Abrahamsson A., Blum J., Kwon H.Y., Kim J., Chute J.P., Rizzieri D., Munchhof M., VanArsdale T., Beachy P.A., Reya T. (2009). Hedgehog signaling is essential for maintenance of cancer stem cells in myeloid leukaemia. Nature.

[18] Guzman M.L., Rossi R.M., Karnischky L., Li X.J., Peterson D.R., Howard D.S., Jordan C.T. (2005). The sesquiterpene lactone parthenolide induces apoptosis of human acute myelogenous leukemia stem and progenitor cells. Blood.

[19] Ji Q., Ding Y.H., Sun Y., Zhang Y., Gao H.E., Song H.N., Yang M., Liu X.L., Zhang Z.X., Li Y.H., Gao Y.D. (2016). Antineoplastic effects and mechanisms of micheliolide in acute myelogenous leukemia stem cells. Oncotarget.

[20] Zhang Q., Lu Y.X., Ding Y.H., Zhai J.D., Ji Q., Ma W.W., Yang M., Fan H.X., Long J., Tong Z.S., Shi Y.H., Jia Y.S., Han B., Zhang W.P., Qiu C.J., Ma X.Y., Li Q.Y., Shi Q.Q., Zhang H.L., Li D.M., Zhang J., Lin J.P., Li L.Y., Gao Y.D., Chen Y. (2012). Guaianolide sesquiterpene lactones, a source to discover agents that selectively inhibit acute myelogenous leukemia stem and progenitor cells. J. Med. Chem..

[21] Ding Y.H., Gao H.E., Zhang Y., Li Y., Vasdev N., Gao Y.D., Chen Y., Zhang Q. (2016). Alantolactone selectively ablates acute myeloid leukemia stem and progenitor cells. J. Hematol. Oncol..

[22] Guzman M.L., Li X.J., Corbett C.A., Rossi R.M., Bushnell T., Liesveld J.L., Hebert J., Young F., Jordan C.T. (2007). Rapid and selective death of leukemia stem and progenitor cells induced by compound 4-benzyl, 2-methyl, 1,2,4-thiadiazolidine,3,5 dione (TDZD-8). Blood.

[23] Gao M., Wang X.Z., Shu Y.T., Liang J., Chen L., Liu R., Liang J.Y., Wen H.M. (2017). Peperomin E and its synthetic amino derivatives: potent agents targeting leukaemia stem cells. RSC Adv..

[24] Arner E.S.J., Zhong L.W., Holmgren A. (1999). Preparation and assay of mammalian thioredoxin and thioredoxin reductase. M. Enzynol..

[25] Huang L., Chen Y., Liang B.X., Xing B.G., Wen G.S., Wang S.N., Yue X., Zhu C.G., Du J., Bu X.Z. (2014). A furanyl acryl conjugated coumarin as an efficient inhibitor and a highly selective off-on florescent probe for covalent labelling of thioredoxin reductase. Chem. Commun..

[26] Fang J.G., Lu J., Holmgren A. (2005). Thioredoxin reductase is irreversibly modified by curcumin. J. Biol. Chem..

[27] Kaminska K.K., Bertrand H.C., Tajima H., Stafford W.C., Cheng Q., Chen W., Wells G., Arner E.S.J., Chew E.H. (2015). Indolin-2-one compounds targeting thioredoxin reductase as potential anticancer leads. Oncotarget.

[28] Tuladhar A., Rein K.S. (2018). Manumycin A is a potent inhibitor of mammalian thioredoxin reductase-1. ACS Med. Chem. Lett..

[29] Duan D.Z., Zhang J.M., Yao J., Liu Y.P., Fang J.G. (2016). Targeting thioredoxin reductase by parthenolide contributes to inducing apoptosis of Hela cells. J. Biol. Chem..

[30] Liu Y., Duan D., Yao J., Zhang B., Peng S., Ma H.L., Song Y.L., Fang J.G. (2014). Dithiaarsanes induce oxidative stress-mediated apoptosis in HL-60 cells by selectively targeting thioredoxin reductase. J. Med. Chem..

[31] Wang X.Z., Wen H.M., Chai C., Zhang W.Y., Gao M., Liu R., Wu H., Liang J.Y. (2017). Determination of a natural DNMT1 inhibitor, peperomin E, in rat plasma by UFLC-MS/MS and method application in a pharmacokinetic study. Biomed. Chromatogr..

[32] Shi X.K., Zhang Y., Zheng J.H., Pan J.X. (2012). Reactive oxygen species in cancer stem cells. Antioxid. Redox Signal.

[33] Kobayashi C.I., Suda T. (2011). Regulation of reactive oxygen species in stem cells and cancer stem cells. J. Cell. Physiol..

[34] Pei S.S., Minhajuddin M., Callahan K.P., Balys M., Ashton J.M., Neering S.J., Lagadinou E.D., Corbett C., Ye H.B., Liesveld J.L., O'Dwyer K.M., Li Z., Shi L., Greninger P., Settleman J., Benes C., Hagen F.K., Munger J., Crooks P.A., Becker M.W., Jordan C.T. (2013). Targeting aberrant glutathione metabolism to eradicate human acute myelogenous leukemia cells. J. Biol. Chem..

[35] Zhang J.M., Li X.M., Han X., Liu R.J., Fang J.G. (2017). Targeting the thioredoxin system for cancer therapy. Trends Pharmacol. Sci..

[36] Yang Y., Chen Y., Johansson E., Schneider S.N., Shertzer H.G., Nebert D.W., Dalton T.P. (2007). Interaction between the catalytic and modifier subunits of glutamate-cysteine ligase. Biochem. Pharmacol..

[37] Sunde R.A., Evenson J.K. (1987). Serine incorporation into the selenocysteine moiety of glutathione peroxidase. J. Biol. Chem..

[38] Griffith O.W. (1999). Biologic and pharmacologic regulation of mammalian glutathione synthesis. Free Rad. Biol. Med..

[39] Duan D.Z., Zhang B.X., Yao J., Liu Y.P., Fang J.G. (2014). Shikonin targets cytosolic thioredoxin reductase to induce ROS-mediated apoptosis in human promyelocytic leukemia HL-60 cells. Free Rad. Biol. Med..

[40] Lee S.R., Bar-Noy S., Kwon J., Levine R.L., Stadtman T.C., Rhee S.G. (2000). Mammalian thioredoxin reductase: oxidation of the C-terminal cysteine/selenocysteine active site forms a thioselenide, and replacement of selenium with sulfur markedly reduces catalytic activity. Proc. Nat. Acd. Sci..

[41] Anestal K., Prast-Nielsen S., Cenas N., Arner E.S.J. (2008). Cell death by SecTRAPs: thioredoxin reductase as a prooxidant killer of cells. PLoS One.

[42] Saitoh M., Nishitoh H., Fujii M., Takeda M., Tobiume K., Sawada Y., Kawabata M., Miyazono K., Ichijo H. (1998). Mammalian thioredoxin is a direct inhibitor of apoptosis signal-regulating kinase (ASK) 1. EMBO J..

[43] Yan C., Siegel D., Newsome J., Chilloux A., Moody C.J., Ross D. (2012). Antitumor indolequinones induced apoptosis in human pancreatic cancer cells via inhibition of thioredoxin reductase and activation of redox signaling. Mol. Pharmacol..

[44] Zou P., Xia Y.Q., Ji J.S., Chen W.Q., Zhang J.S., Chen X., Rajamanickam V., Chen G.Z., Wang Z., Chen L.F., Wang Y.F., Yang S.L., Liang G. (2016). Piperlongumine as a direct TrxR1 inhibitor with suppressive activity against gastric cancer. Cancer Lett..

[45] Cai W.Q., Zhang L.W., Song Y.L., Wang B.L., Zhang B.L., Cui X.M., Hu G.M., Liu Y.P., Wu J.C., Fang J.G. (2012). Small molecule inhibitors of mammalian thioredoxin reductase. Free Rad. Biol. Med..

