# Supplementary material for: Peperomin E and its orally bioavailable analog induce oxidative stress-mediated apoptosis of acute myeloid leukemia progenitor cells by targeting thioredoxin reductase
Source: Redox Biol. 2019 Mar 8;24:101153. doi: 10.1016/j.redox.2019.101153 (PMC6434189; doi:10.1016/j.redox.2019.101153)
Supplement: Multimedia component 2 [file mmc2.docx]

***Supplementary Materials***

**Fig. S1.** ^1^H-NMR (a) and ^13^C-NMR (b) spectrum of *Peperomin E* (PepE)

**Fig.S2.** ^1^H-NMR (a) and ^13^C-NMR (b) spectrum of *(2S,3S)-6-Methyl (hydroxyethyl) amino-2,6-dihydropeperomin E* (DMAPE)

**Fig. S3.** ^1^H-NMR (a) and ^13^C-NMR (b) spectrum of *Peperomin A* (PepA)

**Table S1.** Clinical information of primary human AML speciemens

**Table S2.** Primer list for RT-qPCR study

**Table S3.** Kinetic analysis of the interaction between purified recombinant human antioxidant proteins and PepE by BLI assay

**Additional Method.** Experimental method for the pharmacokinetic study of DMAPE

**List of abbreviations in the manuscript**


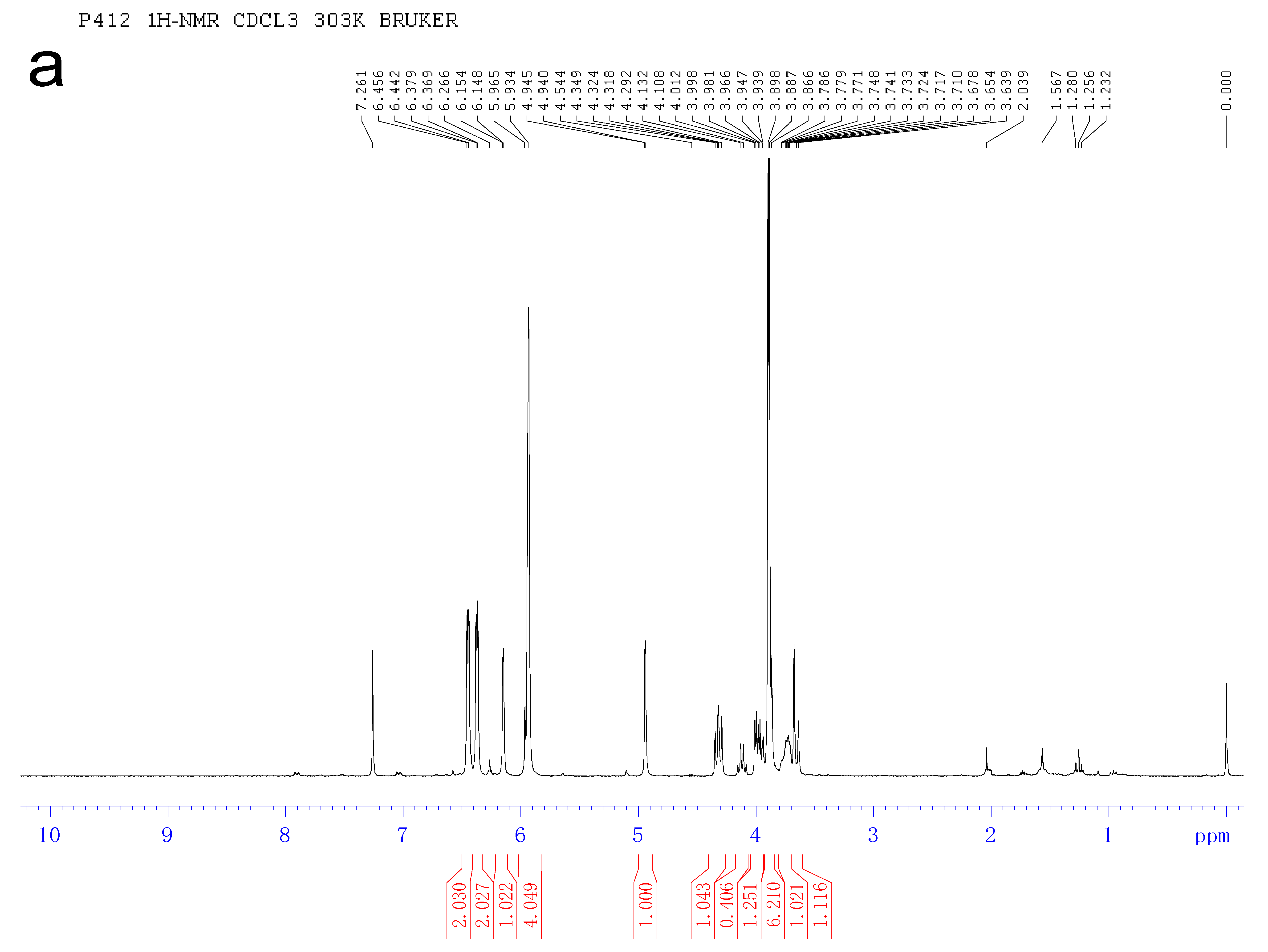


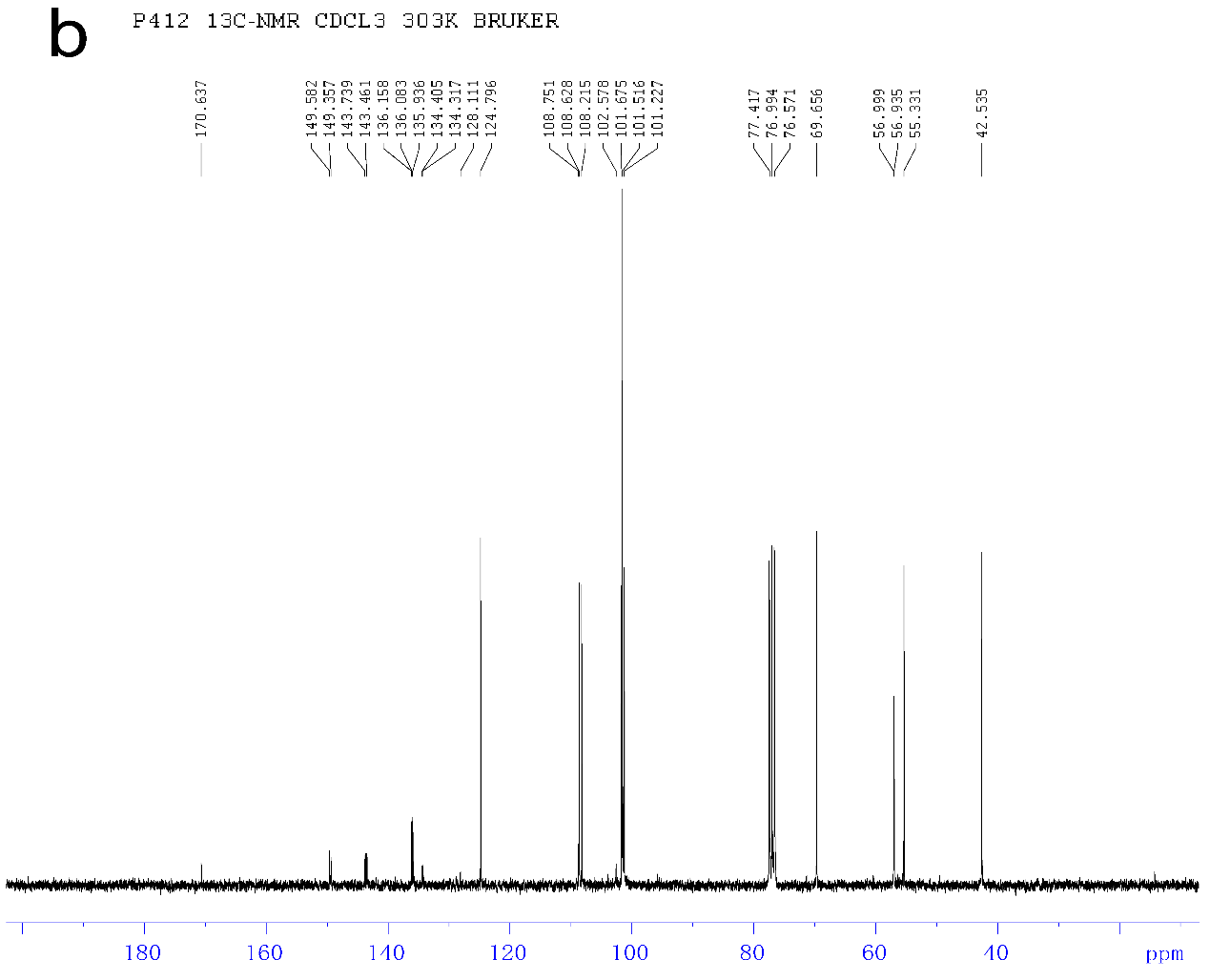


**Fig S1.** ^1^H-NMR (a) and ^13^C-NMR (b) spectrum of Peperomin E (PepE)

**Peperomin E (PepE)**

**
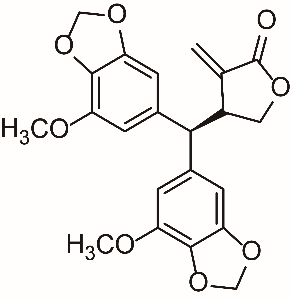
**

Colourless powder; 98.0% HPLC purity; **^1^H-NMR (500MHz, CDCl_3_)** *δ*_H_ 6.456, 6.443 (*s*, 2H）, 6.379, 6.369(*s*, 2H), 6.151(*d*, *J*=1.8Hz, 1H), 5.934(*s*, 4H), 4.943(*d*, *J*=1.5Hz, 1H), 4.321(*dd*, *J*=7.5, 9.3Hz, 1H), 3.940(*dd*, *J*=4.2, 9.3Hz, 1H), 3.887(*s*, 3H), 3.897(*s,*3H), 3.74(*m*, 1H). **^13^C NMR (125 MHz, CDCl_3_)** *δ*_C_ 170.4, 135.9, 124.7, 134.4, 134.3, 149.4, 149.6, 143.7, 143.5, 136.1, 136.2, 108.2, 108.4, 101.7, 101.2, 101.5, 42.5, 55.3, 56.9, 57.0, 69.7 ppm. **HRMS-ESI calcd for** C_22_H_20_O_8_Na [M+Na]^+^ 435.1056,found 435.1041.

**a**


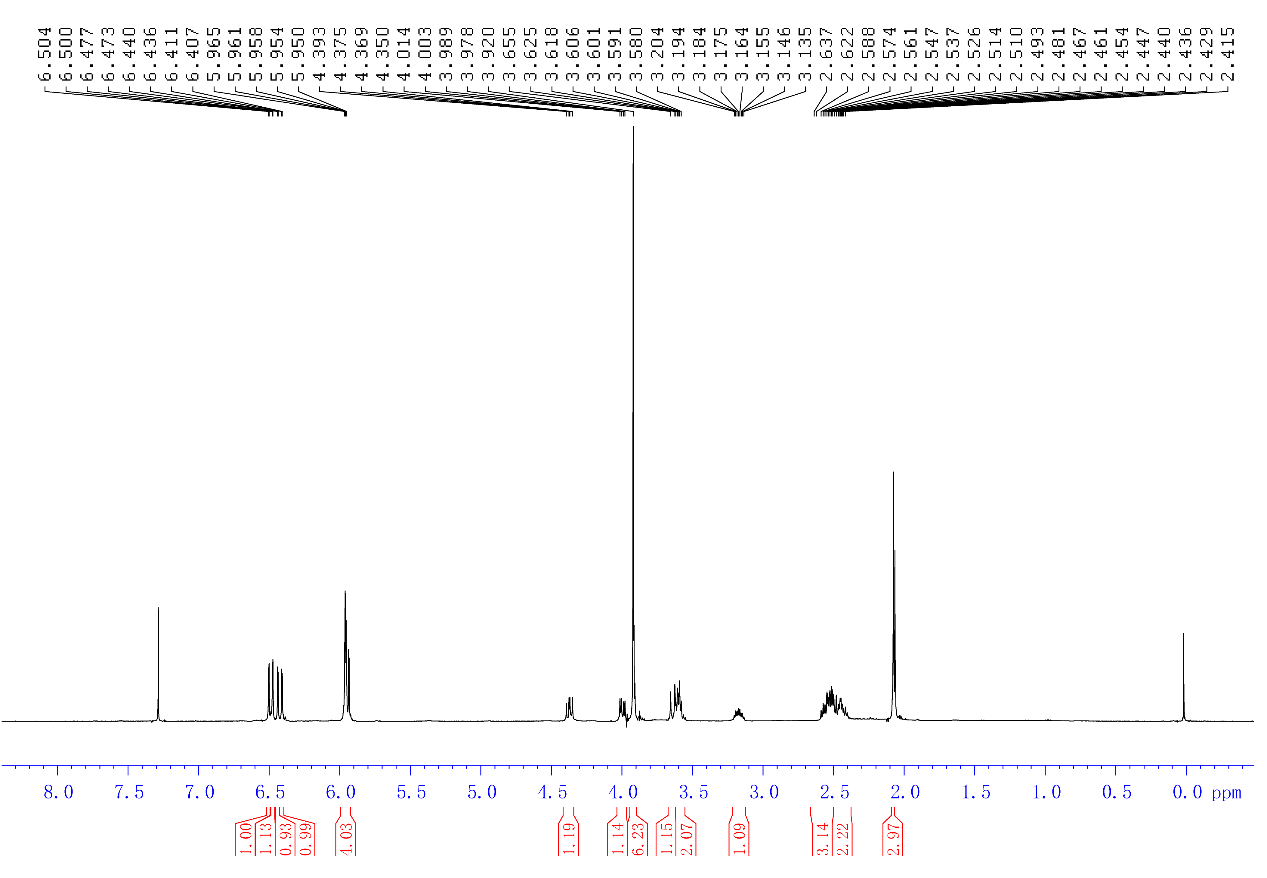


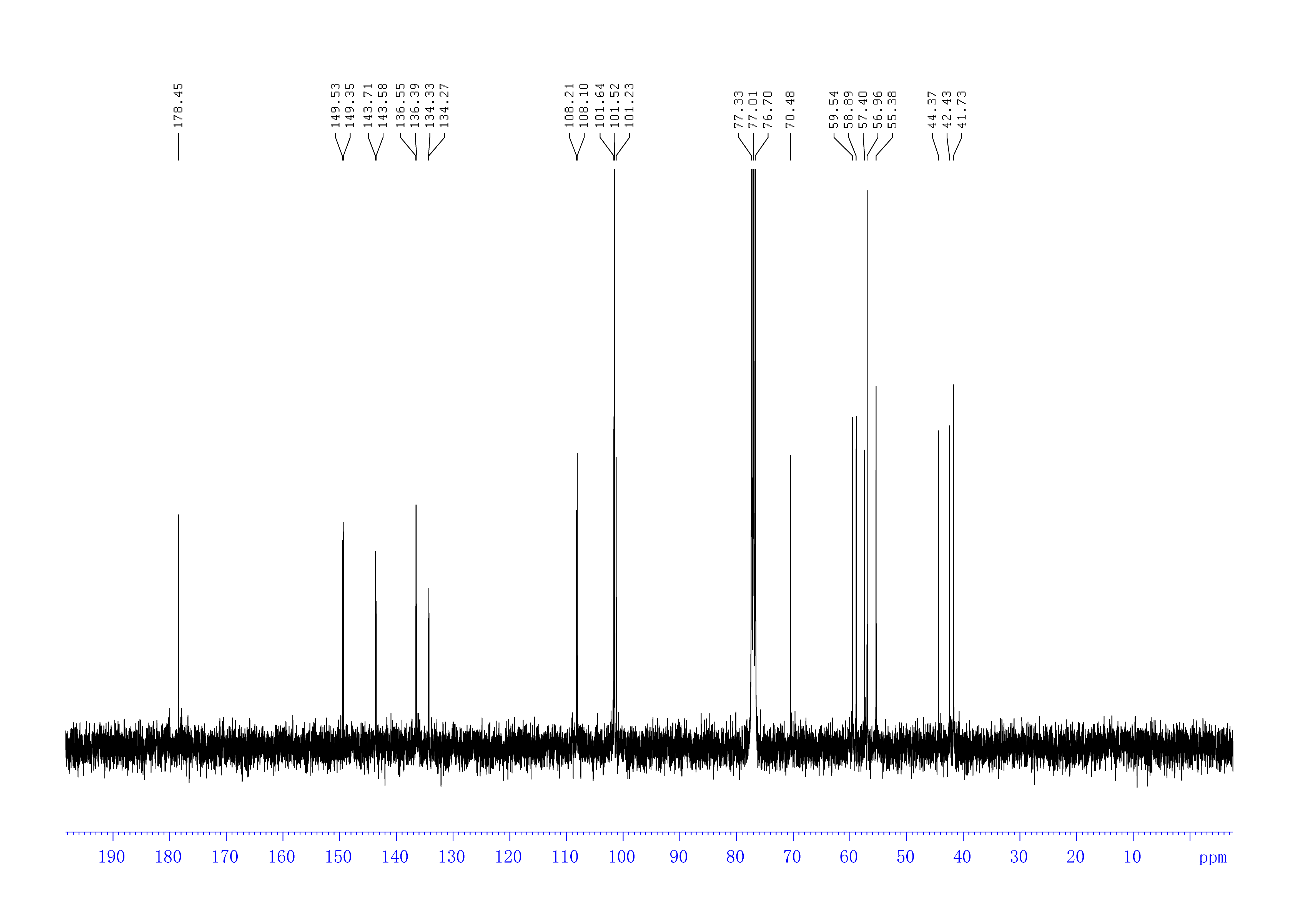


**b**

**Fig S2.** ^1^H-NMR (a) and ^13^C-NMR (b) spectrum of *(2S,3S)-6-Methyl (hydroxyethyl) amino-2,6-dihydropeperomin E* (DMAPE)

***(2S,3S)-6-Methyl(hydroxyethyl)amino-2,6-dihydropeperomin E* (DMAPE)**

***
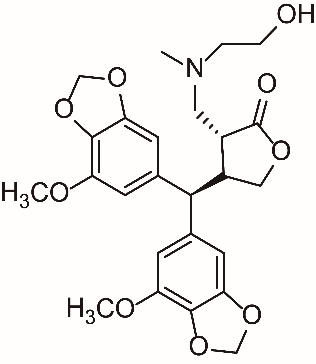
***

Colourless powder;100.0% HPLC purity; **IR (thin film)** *ν*_max_ 3445.62, 2917.98, 1767.13, 1633.65, 1507.61, 1451.75, 1435.25, 1192.73, 1135.07, 1090.75, 1042.05, 927.53, and 852.33 cm^-1^; **^1^H NMR (500MHz, CDCl_3_)** *δ*_H_ 6.50 (*d*, *J*=2.0 Hz, 1H), 6.48 (*d*, *J*=2.0 Hz, 1H), 6.44 (*d*, *J*=2.0 Hz, 1H), 6.41 (*d*, *J*=2.0 Hz, 1H), 5.95-5.97 (*m*, 4H), 4.37 (*dd*, *J*=9.0, 12.0 Hz, 1H), 4.00 (*dd*, *J*=5.5, 12.0 Hz, 1H), 3.92 (*s*, 6H), 3.64 (*d*, *J*=15.5 Hz, 1H), 3.62-3.58 (*m*, 2H), 3.14-3.20 (*m*, 1H), 2.54-2.64 (*m*, 3H), 2.42-2.53 (*m*, 2H), 2.08 (*s*, 3H); **^13^C NMR (125 MHz, CDCl_3_)** *δ*_C_ 178.5, 149.5, 149.3, 143.7, 143.6, 136.6, 136.4, 134.3 (x2), 108.2, 108.1, 101.6, 101.5, 101.2 (x2), 70.5, 59.5, 58.9, 57.4, 56.9 (x2), 55.4, 44.4, 42.4, and 41.7 ppm; **HRMS-ESI** calcd for C_25_H_30_NO_9_ [M+H]^+^ 488.1921, found 488.1890.


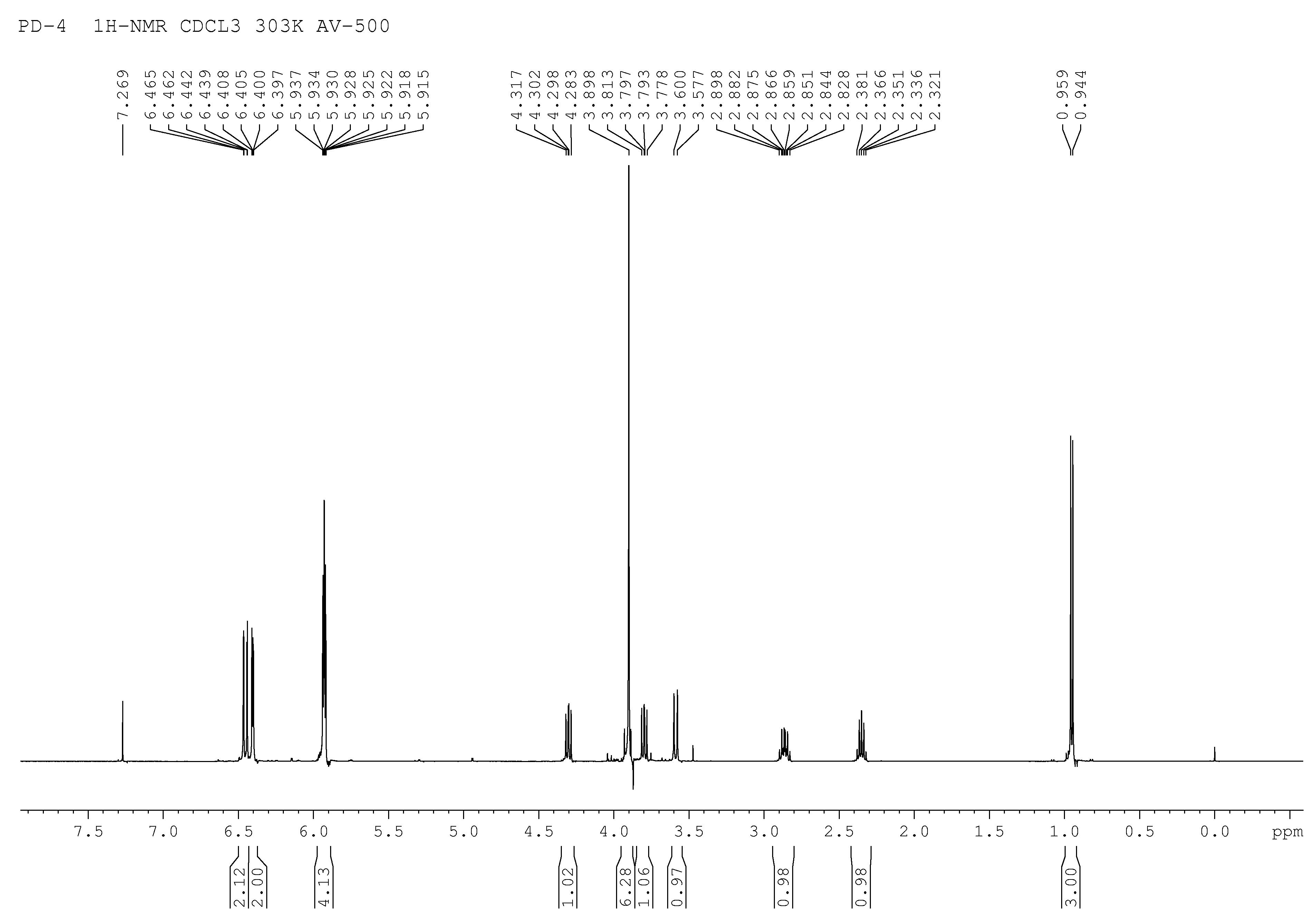


**b**

**a**


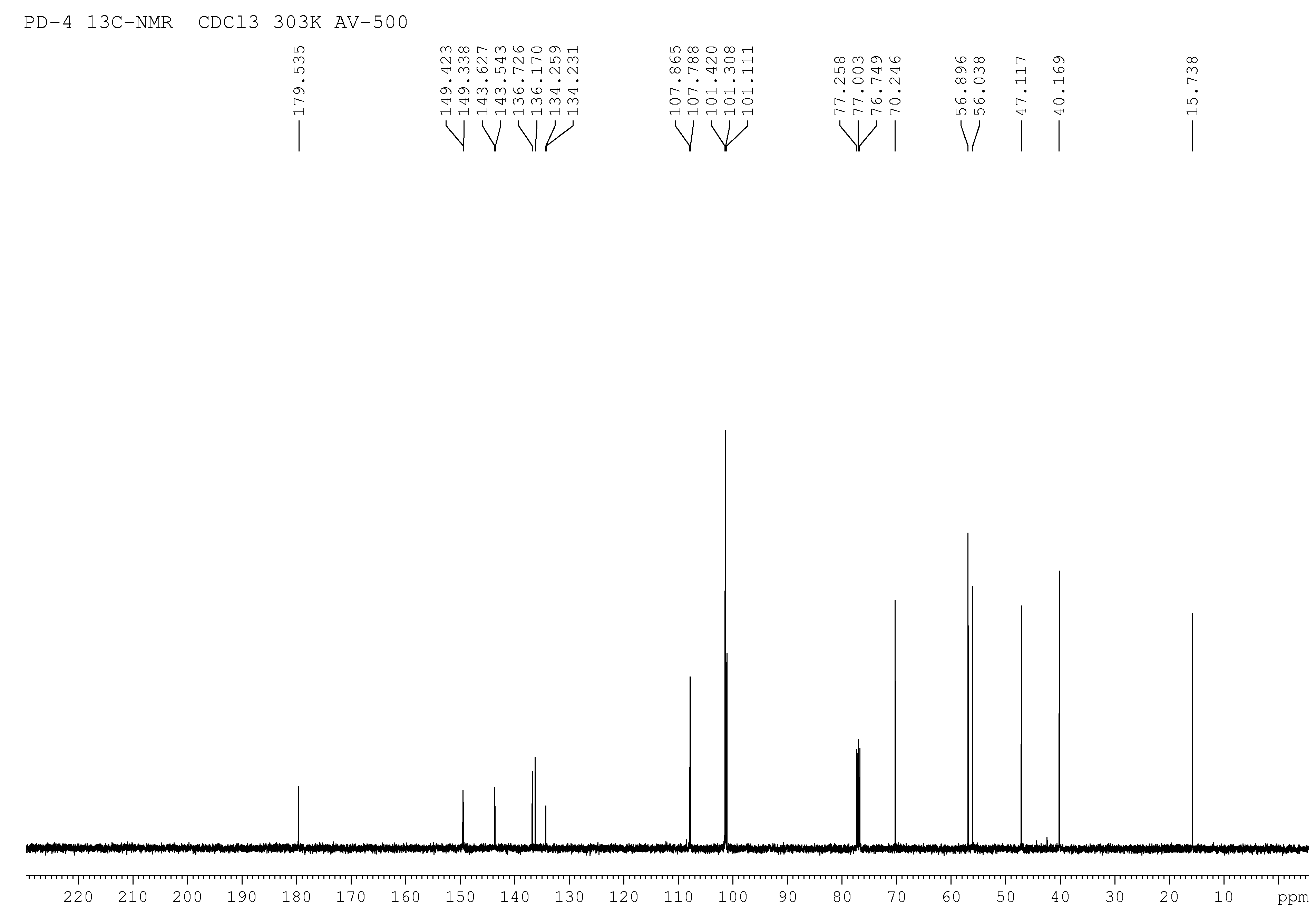


**Fig S3.** ^1^H-NMR (a) and ^13^C-NMR (b) spectrum of *Peperomin A* (PepA)

**Peperomin A (PepA)**

Colourless powder;99.5% HPLC purity; **^1^H NMR (500MHz, CDCl_3_)** *δ*_H_ 6.46 (*d*, *J*=1.5Hz, 1H), 6.44 (*d*, *J*=1.5 Hz, 1H), 6.41 (*d*, *J*=1.5 Hz, 1H), 6.40 (*d*, *J*=1.5 Hz, 1H), 5.93 (*m*, 4H), 4.30 (*dd*, *J*=8.0, 10.0 Hz, 1H), 3.90 (*s*, 6H), 3.79 (*dd*, *J*=8.0, 10.0 Hz, 1H), 3.59 (*d*, *J*=11.5 Hz, 1H), 2.86 (*m*, 1H), 2.35 (*m*, 1H), 0.95 (*d*, 3H, *J*=7.2 Hz ); **^13^C NMR (125 MHz, CDCl_3_)** *δ*_C_ 179.5, 149.4, 149.3, 143.6, 143.5, 136.7, 136.2, 134.4, 134.2, 107.9, 107.8, 101.3, 101.4, 101.1, 70.3, 56.9 (X2), 56, 47.1, 40.2, and 15.7 ppm; **HRMS-ESI** calcd for C_22_H_23_O_8_ [M+H]^+^ 415.1393, found 415.1391.

**Table S1**

Clinical information of primary human AML specimens

| **Specimen** | **Gender** | **Age** | **FAB^a^** | **Diagnosis** | **Cytogenetics^b^** | **Mutations^c^** | **Immuno-phenotyping^d^** |
| --- | --- | --- | --- | --- | --- | --- | --- |
| ***AML1*** | Male | 42 | M1 | *De novo* AML | Normal | FLT3-ITD^+^ | CD34^+^, HLA-DR^+^, CD33^+^,CD13^+^ |
| ***AML2*** | Female | 63 | M1 | *De novo* AML | t(6; 9) | None | HLA-DR^+^, CD33^+^,CD13^+^ |
| ***AML3*** | Male | 55 | M1 | Relapsed AML | Normal | FLT3-ITD^+^ | CD34^+^, HLA-DR^+^, CD33^+^,CD13^+^ |
| ***AML4*** | Male | 61 | M1 | Relapsed AML | Normal | FLT3-ITD^+^,  NPM1^+^ | CD34^+^, HLA-DR^+^, CD33^+^,CD13^+^ |
| ***AML5*** | Female | 48 | M1 | Relapsed AML | Normal | FLT3-ITD^+^ | CD34^+^, HLA-DR^+^, CD33^+^,CD13^+^ |

1. French-American-British AML subtype classification.
2. Cytogenetic studies were performed using the standard protocol of Giemsa banding analysis at Jiangsu Province Hospital on Integration of Chinese and Western Medicine, and chromosomal abnormalities were described according to the International System for Cytogenetic Nomenclature.
3. The FLT3-ITD and NPM1 mutations were detected by PCR analysis using the standard protocol of Jiangsu Province Hospital on Integration of Chinese and Western Medicine.
4. Immunophenotyping detection was performed using the standard protocol of flow cytometry analysis at Jiangsu Province Hospital on Integration of Chinese and Western Medicine.

**Table S2**

Primer list for RT-qPCR study

| **Gene Symbol** | **Sense 5'-3'** | **Antisense 5'-3'** | **Cycles** | **Annealing temperature (^o^C)** |
| --- | --- | --- | --- | --- |
| ***h-SOD1*** | AGGCCCCTTAACTCATCT | CTACAGGTACTTTAAAGCAACTCT | 25 | 55 |
| ***h-SOD2*** | AAGGGAGATGTTACAGCCCAGATA | TCCAGAAAATGCTATGATTGATATGAC | 30 | 55 |
| ***h-GCLC*** | TTGAGGCCAACATGCGAAA | AGGACAGCCTAATCTGGGAAATG | 25 | 60 |
| ***h-GSS*** | AGCGTGCCATAGAGGAATGAG | ATCCCGGAAGTAAACCACAG | 30 | 60 |
| ***h-CAT*** | TTTCCCAGGAAGATCCTGAC | ACCTTGGTGAGATCGAATGG | 30 | 60 |
| ***h-GPX1*** | CCCTCTGAGGCACCACGGT | TAAGCGCGGTGGCGTCGT | 20 | 60 |
| ***h-GSR*** | CAGTGGGACTCACGGAAGA | TTCACTGCAACAGCAAAACC | 30 | 55 |
| ***h-PRX1*** | ATGTCTTCAGGAAATGCTAAAAT | ACTTCTGCTTGGAGAAATATTC | 35 | 60 |
| ***h-TrxR1*** | AATGTACTGGCTGAGGATTC | ATGAGATGAGGACGTGAGGC | 25 | 55 |
| ***h-HPRT1*** | TGAGGATTTGGAAAGGGTGT | GAGCACACAGAGGGCTACAA | 25 | 55 |
| ***h-GAPDH*** | CCTCTGACTTCAACAGCGACAC | CTGTTGCTGTAGCCAAATTCGT | 30 | 60 |

**Table S3.**

Kinetic analysis of the interaction between purified recombinant human antioxidant proteins and PepE by BLI assay

| **Proteins** | **K_on_**  **(1/Ms)** | **K_dis_**  **(1/s)** | **K_D_**  **(M)** | **Full R^2^** |
| --- | --- | --- | --- | --- |
| ***GPX1*** | 6.40×10^3^ | 3.42×10^1^ | 5.34×10^-4^ | 0.98 |
| ***GSS*** | 2.74×10^3^ | 7.45×10^-1^ | 2.72×10^-4^ | 0.92 |
| ***TrxR1*** | 1.24×10^4^ | 4.23×10^-3^ | 3.41×10^-7^ | 0.90 |
| ***GCLC*** | 4.65×10^2^ | 6.25×10^-1^ | 1.34×10^-3^ | 0.90 |

***Additional Method-***

**Experimental method for the pharmacokinetic study of DMAPE**

***1. UFLC–MS/MS analysis***

Prominence^TM^ UFLC system (Shimadzu, Kyoto, Japan) coupled with a QTRAP^TM^ 5500 MS/MS system (Applied Biosystems/MDS Sciex, Foster City, CA, USA) were used for sample analysis. Separation was achieved on an YMC-Pack Pro C_18_ column (2.0 mm × 50 mm, 3.0 μm) with a guard cartridge(C_18_, 2mm × 4 mm, Phenomenex, Torrance, CA, USA) at a temperature of 30 °C, and elution was performed with an isocratic mobile phase of acetonitrile and 0.1% (v/v) formic acid (40:60, v/v) at a flow rate of 0.4 mL/min.

Analytes were detected using a TurboIon spray source in a positive ionization mode and quantified in a multiple reaction monitoring (MRM) mode. The Q1, Q3, declustering potential (DP), collision energy (CE), and collision cell exit potential (CXP) values were measured using the Analyst 1.5.2 software (Applied Biosystems/MDS SCIEX, Table 1). The other ionization parameters were as follows: curtain gas, 35 (arbitrary units); ion source gas 1 and 2, 55 (arbitrary units); source temperature, 500 °C; and entrance potential, 10 V. The dwell time of each MRM transition was 50 ms. The UFLC-MS/MS system was controlled by the Analyst 1.5.2 software.

**Table 1**

Optimized multiple reaction monitoring (MRM) parameters for the analytes and arctigenin (IS)

| **Analytes** | **Q1/Q3（*m/z*）** | **DP (V)** | **CE (eV)** | **CXP (V)** |
| --- | --- | --- | --- | --- |
| DMAPE | 488.0/88.0 | 126.0 | 20.0 | 10.0 |
| PepE | 413.2/165.1 | 117.0 | 37.0 | 10.0 |
| IS | 372.9/136.9 | 116.0 | 30.0 | 10.0 |

***2. Sample preparation***

The frozen plasma samples were thawed and allowed to equilibrate at ambient temperature before analysis. Then, 10 μL of the IS solution and 1.9 mL of methanol were added to a 100 μL aliquot of rat plasma. After vortex-mixing for 90 s and centrifugation at 12,000 rpm for 10 min, the supernatant was collected and evaporated to dryness at 40 °C using the CentriVap vacuum centrifugal concentrator (Labconco, Kansas City, MO, USA). The resulting residue was reconstituted in 100 μL of methanol, vortexed, and centrifuged at 12,000 rpm for 10 min. A 2 μL aliquot of the supernatant was injected for UFLC–MS/MS analysis.

***3. Method validation***

***3. 1 Selectivity***

Fig 1 shows the chromatograms of drug-free rat plasma, blank plasma spiked with IS, DMAPE and PepE at their LLOQ concentration. No interfering endogenous substance was observed at the retention times of DMAPE, PepE and IS.





**Fig 1.** Representative MRM chromatograms of DMAPE, PepE and arctigenin (IS) in rat plasma at their LLOQ concentrations.

***3.2 Linearity and LLOQ***

The calibration curve were linear over concentration ranges of 2.0-2,000 ng/mL for DMAPE, 5.0-2,000 ng/mL for PepE. The regression equations obtained by weighted (1/*x^2^*) least squares linear regression were *y = 0.1322x - 0.3799* (*r*^2^ = 0.9999) for DMAPE and *y = 0.0227x + 0.1084* (*r^2^* = 0.9999), respectively, where *y* represents the peak-area ratio of DMAPE/PepE to IS and *x* stands for the plasma concentration of DMAPE/PepE. The LLOQ for DMAPE/PepE in rat plasma were established to be 2.0 and 5.0 ng/mL, respectively.

***3.3 Precision and accuracy***

Intra- and interday precision and accuracy were evaluated at low (25 ng/mL), medium (1,000 ng/mL), and high (5,800 ng/mL) QC concentration levels of both DMAPE and PepE, with six replicates per day on three consecutive days. The accuracy was expressed as a percentage of the mean calculated concentration relative to the spiked concentration. The precision and accuracy data for the determination of DMAPE/PepE at the three QC levels are summarized in Table 2. The relative errors (REs) obtained ranged from −16.5 to 2.4% for the intraday accuracy and from −6.6 to 2.0% for the interday accuracy, with the RSD value of less than 8.5%, which indicated that the method was acceptable.

**Table 2.**

Precision and accuracy for the analytes in rat plasma

| **Analytes** | **Days** | **Batches** | **25**  **ng/mL** | **1,000**  **ng/mL** | **5,000**  **ng/mL** |
| --- | --- | --- | --- | --- | --- |
| DMAPE | D1 | RSD% | 7.1% | 8.5% | 2.5% |
|  |  | RE% | 2.4% | -0.9% | -5.0% |
|  | D2 | RSD% | 3.8% | 2.5% | 2.6% |
|  |  | RE% | -16.5% | -7.5% | -3.7% |
|  | D3 | RSD% | 6.9% | 1.4% | 1.7% |
|  |  | RE% | -2.6% | -9.3% | -7.5% |
|  | Inter-Day | RSD% | 1.9% | 0.9% | 1.0% |
|  |  | RE% | 2.0% | -1.1% | -3.6% |
| Pep E | D1 | RSD% | 4.1% | 6.9% | 5.1% |
|  |  | RE% | -3.9% | -4.6% | -5.4% |
|  | D2 | RSD% | 3.7% | 2.9% | 1.5% |
|  |  | RE% | -6.4% | -1.9% | -6.1% |
|  | D3 | RSD% | 9.1% | 1.4% | 1.8% |
|  |  | RE% | -1.3% | -3.4% | -4.5% |
|  | Inter-day | RSD% | 7.3% | 1.8% | 1.6% |
|  |  | RE% | 1.9% | -6.6% | -1.8% |

***3.4 Extraction recovery***

Extraction recovery was calculated by comparing pre- and post-extraction peak areas in rat plasma samples spiked with DMAPE and PepE, respectively, at the three QC concentration levels (25, 1,000, and 5,000 ng/mL). The mean extraction recovery of DMAPE from rat plasma at the three different concentration levels was found to be 94.7, 101.7, and 106.8%, with the RSD values of 0.7, 7.1, and 2.7%, respectively, whereas the mean recovery of PepE from rat plasma at the same concentration levels was 89.5%, 86.2% and 84.7%, with the RSD value of 13.5, 6.0 and 5.6%, respectively (*n* = 6). The data, which were well within the acceptable limit, are summarized in Table 3.

**Table 3.**

Extraction recovery for the analytes in rat plasma

| **Analytes** | **Concentration**  **(ng/ml)** | **Recovery Rates (%)** | | | | | | **Mean**  **(%)** | **RSD**  **(%)** |
| --- | --- | --- | --- | --- | --- | --- | --- | --- | --- |
|  |  | **1** | **2** | **3** | **4** | **5** | **6** |  |  |
| **DMAPE** | 5 | 94.9 | 93.7 | 95.4 | 94.2 | 95.5 | 94.6 | 94.7 | 0.7 |
|  | 200 | 94.5 | 101.9 | 105.8 | 111.6 | 101.9 | 91.8 | 101.7 | 7.1 |
|  | 1000 | 105.3 | 111.5 | 108.6 | 105.5 | 106.4 | 103.4 | 106.8 | 2.7 |
| **PepE** | 5 | 94.6 | 98.6 | 73.0 | 80.1 | 85.6 | 105.3 | 89.5 | 13.5 |
|  | 200 | 94.8 | 85.3 | 89.1 | 81.7 | 80.9 | 85.3 | 86.2 | 6.0 |
|  | 1000 | 84.6 | 87.6 | 89.3 | 86.9 | 75.8 | 83.9 | 84.7 | 5.6 |

***3.5 Matrix effect***

The matrix effect was assessed by comparing the peak areas of post-spiked samples to those of neat standards at the corresponding concentrations. The matrix effect for DMAPE at concentrations of 25, 1000, and 5000 ng/mL was determined to be 99.1, 99.8, and 98.4%, with the RSD values of 3.8, 5.9, and 7.5%, respectively (*n* = 6). The matrix effect for PepE at same concentrations was 101.2, 94.6 and 90.2%, with the RSD value of 5.8, 2.4 and 3.6%, respectively (Table 4). Therefore, the matrix effect from rat plasma was negligible for this method.

**Table 4.**

Matrix effects for the analytes in rat plasma

| **Analytes** | **Concentration**  **(ng/ml)** | **Matrix Effect (%)** | | | | | | **Mean**  **(%)** | **RSD**  **(%)** |
| --- | --- | --- | --- | --- | --- | --- | --- | --- | --- |
|  |  | **1** | **2** | **3** | **4** | **5** | **6** |  |  |
| MADPP | 5 | 95.9 | 104.7 | 98.1 | 100.9 | 100.6 | 94.3 | 99.1 | 3.8 |
|  | 200 | 102.2 | 99.5 | 94.1 | 97.2 | 110.3 | 95.8 | 99.8 | 5.9 |
|  | 1000 | 98.1 | 104.3 | 97.1 | 89.5 | 92.1 | 109.3 | 98.4 | 7.5 |
| Pep E | 5 | 94.8 | 95.9 | 99.5 | 100.3 | 107.7 | 108.8 | 101.2 | 5.8 |
|  | 200 | 96.8 | 92.0 | 93.9 | 97.2 | 92.2 | 95.4 | 94.6 | 2.4 |
|  | 1000 | 92.8 | 89.2 | 89.4 | 85.7 | 95.1 | 89.2 | 90.2 | 3.6 |

***3.6 Stability***

Stability of DMAPE and PepE during sample storage and preparation was determined by analysis of samples at the three concentration levels (25, 1000, and 5000 ng/mL). The results indicated that both DMAPE and PepE were stable in rat plasma samples after storage at −20 °C for 15 days and after three freeze/thaw cycles, as well as at room temperature for 6 h and in an autosampler for processed samples at 4 °C for 12 h (Table 5).

**Table 5.**

Stability of the analytes in rat plasma (n=6)

| Analytes | Concentration  (ng/ml) | Store at  auto-sampler | | Store at -20℃  for 24h | | Freeze-thaw  3 cycles | |
| --- | --- | --- | --- | --- | --- | --- | --- |
|  |  | RSD (%) | RE (%) | RSD(%) | RE (%) | RSD (%) | RE (%) |
| DMAPE | 5 | 7.2 | -11.3 | 6.2 | -11.0 | 5.9 | -13.1 |
|  | 200 | 3.9 | -1.3 | 3.3 | 3.0 | 3.6 | 11.2 |
|  | 1000 | 1.8 | -3.7 | 4.7 | 3.3 | 4.4 | -5.1 |
| Pep E | 5 | 11.4 | -10.1 | 8.4 | 4.4 | 8.7 | -11.5 |
|  | 200 | 5.0 | -10.5 | 3.0 | 11.9 | 1.7 | -13.2 |
|  | 1000 | 1.5 | -7.2 | 5.9 | 1.7 | 0.9 | -3.6 |
